# Proteomic Analysis of Cyclic Ketamine Compounds Ability to Induce Neural Differentiation in Human Adult Mesenchymal Stem Cells

**DOI:** 10.3390/ijms20030523

**Published:** 2019-01-26

**Authors:** Jerran Santos, Bruce Kenneth Milthorpe, Matthew Paul Padula

**Affiliations:** 1 Advanced Tissue Regeneration & Drug Delivery Group, School of Life Sciences, University of Technology Sydney, P.O. Box 123 Broadway, Ultimo 2007, Australia; Bruce.Milthorpe@uts.edu.au; 2 Proteomics Core Facility and School of Life Sciences, Faculty of Science, University of Technology Sydney, P.O. Box 123 Broadway, Ultimo 2007, Australia; Matthew.Padula@uts.edu.au; 3 CIRIMAT, Paul Sabatier, University of Toulouse 3 (INPT), 118 Route de Narbonne, 31062 Toulouse, France

**Keywords:** adult stem cells, adipose, neural, proteomics, cytokines, cyclic ketamine

## Abstract

Neural regeneration is of great interest due to its potential to treat traumatic brain injuries and diseases that impact quality of life. Growth factor mediated differentiation can take up to several weeks to months to produce the cell of interest whereas chemical stimulation may be as minimal as a few hours. The smaller time scale is of great clinical relevance. Adipose derived stem cells (ADSCs) were treated for up to 24 h with a novel differentiation media containing the cyclic ketamine compounds to direct neurogenic induction. The extent of differentiation was investigated by proteome changes occurring during the process. The treatments indicated the ADSCs responded favorably to the neurogenic induction media by presenting a number of morphological cues of neuronal phenotype previously seen and a higher cell population post induction compared to previous studies. Furthermore, approximately 3500 proteins were analyzed and identified by mass spectrometric iTRAQ analyses. The bioinformatics analyses revealed hundreds of proteins whose expression level changes were statistically significant and biologically relevant to neurogenesis and annotated as being involved in neurogenic development. Complementing this, the Bioplex cytokine assay profiles present evidence of decreased panel of stress response cytokines and a relative increase in those involved in neurogenesis.

## 1. Introduction

Stem cell sciences have advanced to the point where it is now possible to provide a range of tissues and cell types to be used in transplantation for regenerative therapies for a wide variety of tissue and organ types [1,2]. However, the production of neural cells from stem cells has been more difficult, and therefore the application of stem cell technology to minimize impairments in neural function or the discovery of compounds capable of the same has been more limited [3]. Prior studies of inducing stem cells toward a neural phenotype most commonly utilized reducing agents or strong antioxidants such as Beta-mercaptoethanol (BME), Dimethylsulfide (DMSO), and Butylated hydroxianisole (BHA) [4,5] or similar compounds. In our previous work [6] these chemicals were implicated as having a strong effect on reduction pathways and decreasing oxidative stress, thus acting as the instigating factor driving the shift from stem cells toward a neural-like phenotype. Krabbe et al. [7], in a review have questioned whether cytotoxic stress is the cause of the neuron-like morphology of MSCs undergoing rapid change. This may well be the case with the above agents. However, if toxicity were the case for the morphological change, one would expect cell stress to show in both the metabolome and proteome overtime.

The work presented here, biologically stable, and non-toxic chemicals with analogous effects on mesenchymal stromal/stem cells (MSCs) to the above-mentioned chemicals have been examined for their potential to drive MSCs towards a neural phenotypic differentiation. The cyclic ketamine (CK) additives; lanthionine ketamine (LK), lanthionine ketamine ethyl ester (LKEE) and *S*-aminoethyl-l-cysteine ketamine (AECK) established the optimal results producing cells that resembled neural cells morphologically. The rationale behind testing these chemicals was that they have a reductive capacity, however, they are non-toxic at the biologically relevant tested concentrations [8].

Briefly, CKs are cyclic sulfur-containing reducing agents that are naturally found in the brain and CNS and have been reported with possessing pro-neural growth properties [8]. CKs basic chemical structure resembles a modified version of the amino acid proline. The two R-groups allow for a wide variety of synthetic and semi-synthetic side chain additions, for example branched-chain alkyl groups and cycloalkyl (alicyclic) groups.

Previous studies with LK and LKEE have shown links to neurotrophic activity, promoting process extension from neurons in vitro that have been shown to protect neurons against oxidative stress [9]. While lanthionine-related compounds have been used for the treatment of inflammatory disease [9] and display antioxidant, neurotrophic, neuroprotective and neuritogenic activity [10], the capacity of these molecules to cause formation of neural cells is unknown. The neurogenic effect on adult stem cells has not been investigated.

This study investigates the proteomic changes of treating stem cells with CK molecules under conditions permitting the formation of a cell having a neural morphological appearance consistent with previous studies. The analysis of the proteome of the differentiated cells gives a deeper insight into the molecular interactions of abundance-regulated proteins during treatment and, critically, whether the cells are neural or simply neural-like with a significant stress response.

## 2. Results

### 2.1. Microscopy

**Figure 1:**
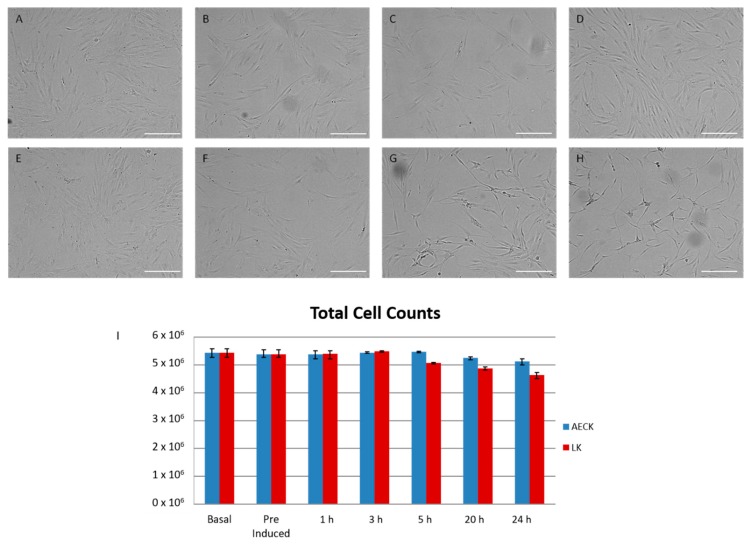
(**A**) Basal human adipose derived stem cells (ADSCs) in vitro culture (non-induced). (**B**–**D**) human ADSCs induced with 0.5 μM AECK time points captured at 3, 5 and 24 h respectively. Time course shows minimal structural changes and some cellular contraction producing cells in (**D**) which are marginally more slender than those in (**A**). (**E**) Basal human ADSCs in vitro culture (non-induced). (**F**–**H**) human ADSCs induced with 0.6 μM lanthionine ketamine (LK) time points captured at 3, 5, and 24 h respectively. Time course adequately exhibits large structural reconfiguration of ADSCs during differentiation. Cells display bipolar elongation, process, and spindle formation. Microscopy images using a 10× objective lens; scale bar 100 μm. (**I**) The cell count for the time course of each treatment AECK (blue) and LK (red).

The treatment of adipose derived stem cells (ADSCs) with the CK and CK derivatives was conducted with the intention of producing cells that morphologically and phenotypically resemble neural cells. A microscopy analysis allowed for the evaluation of the morphological changes produced during the induction. All culture vessels were maintained at sub-confluency prior to addition of differentiation media containing AECK, LK or LKEE. Figure 1A–H shows the rate of cellular response over a 24 h period subsequent to the addition of the novel neurogenic differentiation media. Figure 1B–D are AECK treated ADSCs at time points 3, 5, and 24 h respectively. The AECK treated cells display minimal changes at 3 and 5 h with minor cytoskeletal shifts and retractions. The cells appear to be marginally more slender than the basal cells whilst also producing a condensed nucleus. The final time point exhibited marked changes with a majority of cells assuming a bipolar contouring with a smaller population of approximately 20% showing evidence of process growth and neurite extension similar to primary neural cells [11].

**Figure 2:**
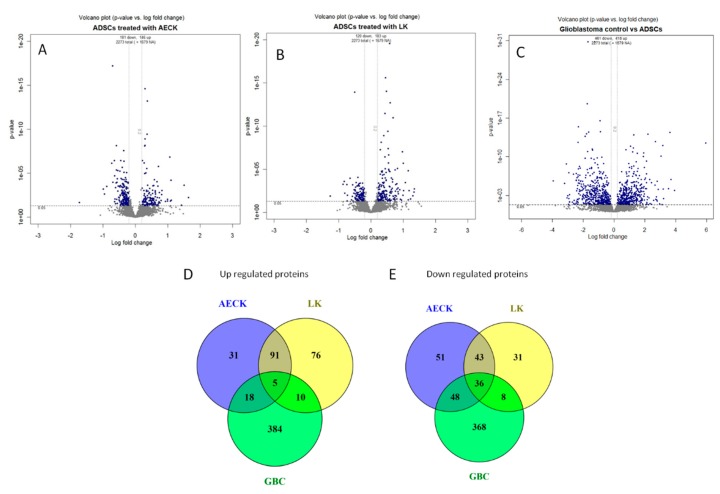
Volcano plots (**A**–**C**) showing *p*-values versus protein fold change (log2) of ADSCs and comparisons generated with DanteR. Quantitation criteria cutoff of statistically significant *p*-values < 0.05 and fold change log2 ratio cutoff of <−0.2 or >0.2. The blue nodes represent the above >0 log fold change, i.e., up-regulated proteins and the below <0 fold change down-regulated proteins. The grey nodes represent the not significantly changed proteins with a *p*-value > 0.05 and within the cut off for fold change. (**D**,**E**) Three-way Venn diagrams of up and down regulated proteins. Diagrams include the AECK treated, LK treated hADSCs, and the GBCs showing unique and shared proteins. (**D**) up regulated proteins revealing there are 31, 76, 384 with 91, 10, 18 shared proteins between each of the corresponding tested cell lines as well as 5 shared proteins between all three relative to basal hADSCs. (**E**) down regulated proteins revealing there are 51, 31, and 368 unique proteins with 43, 8, and 48 shared proteins between each of the corresponding tested cell lines as well as 36 shared proteins between all three relative to basal hADSCs.

The LK treatment produced visually significant morphological changes over time. At the 3-h time point, the cells are indistinguishable from the AECK treatment with minor cytoskeletal retractions toward the nuclei. Beyond the 5-h time point (Figure 1G) the strongest morphological changes from all trialed chemicals is visualized. The LK induced ADSCs are morphologically distinct and bear minimal to no resemblance to the parent basal ADSCs. Cells exhibit elongated membranes and are contracted, displaying a bipolar architecture with multipolar extensions reaching between cells producing visible junctures as seen in Figure 2C. The final time point shows the most expansive and uniform morphological differentiation within this study (Figure 1H). Nearly 95% of cells appear to share the multipolar and dendritic-like extension character in a near network formation. A notable complimentary result is the minimal cell death and low detachment numbers as observed in Figure 2E. The harvested cells at the final time point presented a total dead/live ratio of 1:85; i.e., an average of 1.17% of cells stained blue with trypan, which is 10-fold less than the Beta-mercaptoethanol (BME) treated cells in Santos et al. [6].

### 2.2. iTRAQ Proteome Comparisons of Chemically Induced hADSCs toward Neural Lineage

The digested proteins from each cell line were labelled with the iTRAQ isobaric tags as follows: hADSCs, AECK differentiated hADSCs, LK differentiated hADSCs, and Glioblastoma cells (GBC) were labelled with 114, 115, 116 and 117 isobaric tags respectively. The protein fold changes between samples were done comparatively and are relative to a base denominator, which was the basal hADSCs114, i.e., 115 vs. 114, 116 vs. 114 and 117 vs. 114. This was executed to elucidate the relative protein fold changes across the detected and quantifiable proteome of the differentiating cells, determining the changes in the abundance of protein species over time during differentiation.

Table 1 summarizes the results of the iTRAQ experiment. The summary table shows the upper 99%, 95%, and 66% cut off for significant protein ratio change detected by the iTRAQ quantification. The upper 95% range was chosen for all data analysis and, within that cutoff, the complete analysis had a total of 2430 proteins, consisting of 36,993 distinct peptides identified from 178,574 spectra (protein, peptide, and spectra FDR analysis supplementary material). An average of 6.38 peptides was matched per protein with an average of 18% sequence coverage from the total cohort of the detected proteins (Supplementary Table S1). Proteins were removed from the analysis if they were identified by less than 5 quantifiable peptides/protein to increase the robustness of the dataset and the conclusions drawn. The subsequent cut offs utilized were based on *p*-value (<0.05) and fold change (log2(ratio) less than −0.2 or greater than 0.2). These criteria refine the analysis to statistically significant proteins that have an average of 20 matched peptides per protein, increasing confidence in the data. The ProteinPilot group file, the protein summaries and peptide summary (without background corrections) were exported to XML format for further analysis with specified denominators for inter-sample comparisons through the generation of box and whisker plots, volcano plots and gene ontology graphs in DanteR [12].

Figure 2A–C are the comparative volcano plot layouts of the protein identifications of each iTRAQ labelled ADSC treatment with either AECK or LK and finally the GBC relative to ADSCs. The graphs show the expression differences by fold change (*x*-axis) and statistical significance by *p*-value (*y*-axis) of all captured and identified proteins compared to the basal ADSCs. The blue nodes represent the above >0.2 log fold change up-regulated proteins and the below <0.2 fold change down-regulated proteins. The grey nodes represent the not significantly changed proteins with a *p*-value > 0.05 and within the cut off for fold change. The representation of this data in this format is relevant to assess the range and extent of fold changes and statistical significance occurring across the entire global analysis in a single figure as well as trend comparisons between figures. This also allows for the selection of the most changed proteins in each plot and simultaneous comparison and position selection of the same protein in neighboring plots, thus streamlining comparisons for large iTRAQ datasets. From the volcano plots (Figure 2A–C), Venn diagrams were constructed (Figure 2D,E) representing the number of proteins unique and shared between both replicates for each chemical treatment of the ADSCs. (All the statistically significant proteins are available in Supplementary Table S2.)

### 2.3. Interaction Network Analysis of CK Treated ADSC Proteomes

**Figure 3:**
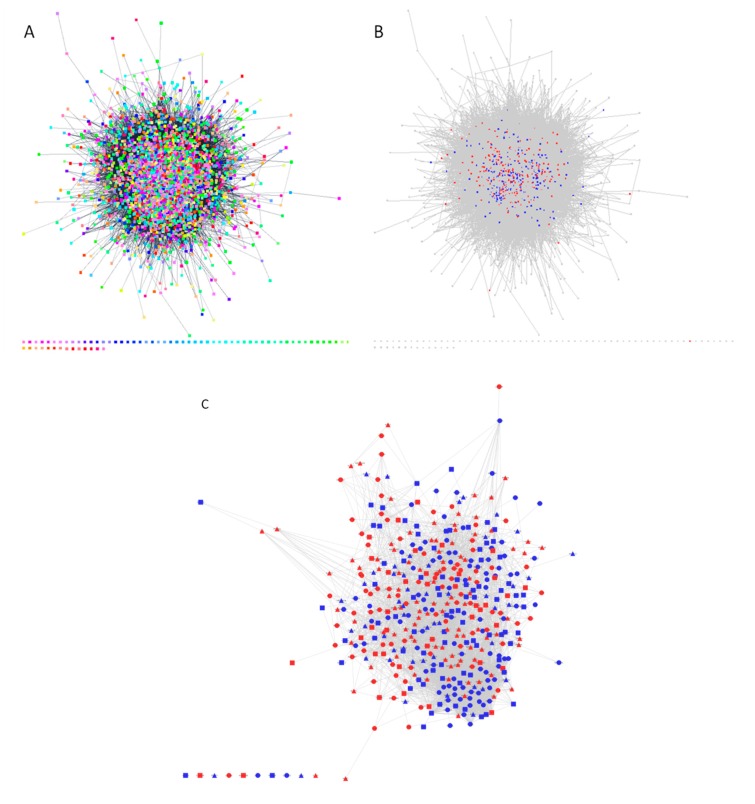

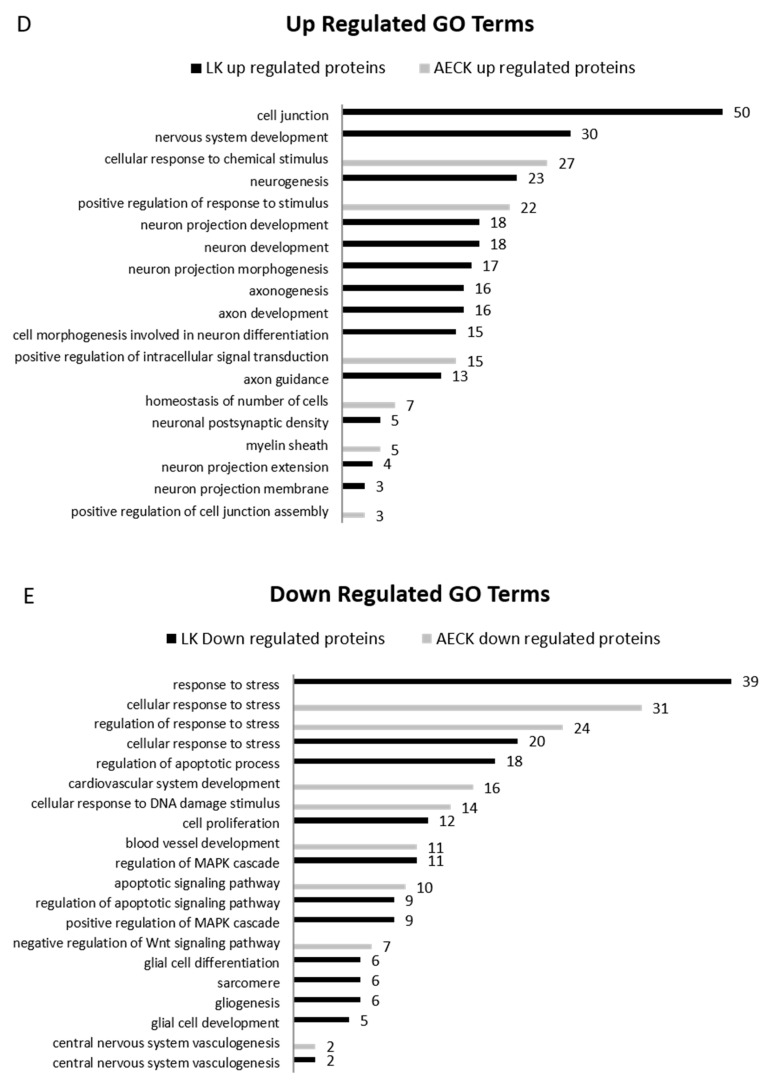
Interaction Networks (**A**) Presents the iTRAQ dataset of 2430 unique proteins nodes each individually colored with a cumulative 90,855 annotated or canonical interactions between proteins across the network presented in grey edge lines. (**B**) Presents the iTRAQ dataset of the full network with all up-regulated protein nodes present in blue and down-regulated protein nodes in red. (**C**) Presents the AECK unique protein nodes as “triangles”, LK protein nodes as squares and shared protein nodes as “circles”. This is in conjunction with the color indication of up regulated proteins in blue and down regulated in red. (**D**,**E**) number of up and down regulated proteins by GO terms respectively.

The open source software Cytoscape [13] works in conjunction with several large annotated databases of protein–protein and protein–DNA interactions that are increasingly available for a variety of organisms. Databases such as Gene Ontology, SwissProt, Ensembl, TrEMBL, UniProt, PDB, EBI, RCSB and STRING are well curated and are easily integrated into the search algorithms. Proteins within the interaction network are represented as nodes and the interactions linking nodes are lines known as edges. The spectrum of edges displayed in networks vary slightly in depth between software; the most common are binding, reaction, catalysis, activation, inhibition, phenotype similarity, post-translational modification, and expression, all of which are sample dependent. Figure 3A is a Cytoscape constructed interaction network from the iTRAQ results representative of the 95% confidence cut off, presenting 2430 unique proteins nodes each individually colored with a cumulative 90,855 annotated or canonical interactions between proteins across the network presented in grey edge lines. To reduce complexity and focus analysis on the ”up” or ”down” regulated proteins, Figure 3B presents the full network with all statistically significant and fold-change criterion cut-off of up-regulated protein nodes present in blue and down-regulated protein nodes in red. Figure 3C displays only the up and down regulation as blue and red respectively, in addition to presenting the protein nodes unique to AECK and LK as well as those that are shared as different symbols. AECK protein nodes are “triangles”, LK protein nodes are “squares” and shared protein nodes are “circles”. Thus displaying, collectively 448 protein nodes with 5425 interaction edges across this subsidiary network. Figure 3D,E presents the relevant up and down regulated proteins from the aforementioned interaction network in Figure 3C as the correlative gene ontology terms. Figure 3D specifies the up regulation of proteins involved in neural differentiation of which a large proportion is involved in neurogenesis and axonogenesis. Complementary to this is Figure 3E which presents the down regulated proteins involved in stress, apoptosis, glial differentiation, and regulation of signaling pathways. Refining this information to particular proteins and their relative biological and statistical significance by abundance and fold changes is presented in Table 2, Table 3, Table 4 and Table 5.

### 2.4. Cytokine Levels

**Figure 4:**
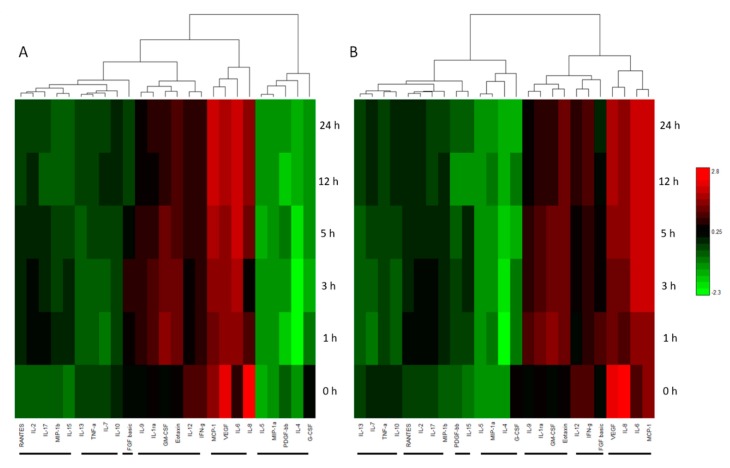
Hierarchical correlation and grouping of up/down regulated cytokines and interleukins secretions from basal ADSCs and temporal differentiation with (**A**) AECK neural differentiation media (**B**) LK neural differentiation media. Hierarchical clustering by Euclidean test Red: expression above median; Green: expression below the median; Black: median expression across all samples.

The Bioplex assay is a multiplex system for investigating the relative quantitative changes of up to 27 cytokines across multiple sample types simultaneously. Section 3.2 covers in detail the relevance of cytokines to neurogenic differentiation. Aliquots of the differentiation media supplemented with either AECK or LK were collected at time points 0, 1, 3, 5, 20 and 24 h and the 27 cytokines measured. An amount per cell (pg/cell) normalization was completed (Supplementary Table S3A,B) to adjust for any discrepancies due to cell death which was apparent in previous studies and, to a limited extent, in the LK and AECK samples. A single tail dendogram heatmap was generated using Euclidean hierarchical clustering for cytokines trends over the differentiation time points (Figure 4A,B). Cytokines have a variety of functions in cellular processes and are often expressed in response to a change in a system which in turn can also regulate the expression of other molecules [14]. Individually and collectively their relative concentrations can be related to particular cellular events or response mechanisms. With this in mind, a number of trends can be observed within the Bioplex temporal ADSC differentiation data set. The comparison of the Bioplex results between AECK and LK treated ADSCs present similar trends with variations within the clustered groups as marked with the hierarchical dendogram. AECK and LK treated ADSCs present 7 notable clustered groups in Figure 4A,B.

## 3. Discussion

The premise of utilizing CK compounds in an attempt to induce ADSCs toward a neural linage was based on several factors pertaining to their chemical properties as well as previous biological observations. Firstly, based on the chemical properties, ketamines are a natural class of sulfur and nitrogen-containing cyclic compounds with reductive activity [15] which are key features of the previously studied simple chemical neurogenic inducing agents, BME and DMSO [6,16]. Furthermore, CK is primarily found in the brain as a natural metabolic by product of the transamination of sulfur-containing amino acids [17,18]. LK and AECK have been purified from bovine brain and binding and interaction studies have been successfully completed in the presence other brain specific imine reductases [19]. LK has also been observed to exhibit neuroprotective, neurotrophic and anti-inflammatory activities [10]. Additionally, the derivative LKEE displayed a higher efficiency for membrane permeability and was revealed to protect motor neurons from oxidative stress in vitro as well as promote neurite outgrowth at nanomolar concentrations [10,20]. The AECK molecule has been noted to react with a similar chemistry to LK and LKEE and is thought to play similar physiological and biochemical roles [21]. The group has also been shown to prevent ischemic neuronal injury via the innate neuroprotective function [22]. CKs are now noted as novel neurotrophic small molecules that hold some promise for the treatment of neurodegenerative diseases [23]. The use of these stable, non-toxic and naturally occurring chemical for promoting the differentiation of a heterogeneous mesenchymal stromal/stem cell population toward a neurogenic lineage was tested and the phenotypic characterization by proteomic quantification was completed.

### 3.1. Neurogenic Related Roles of Identified Proteins

It is now recognized that protein interactions are not random events, their interfaces being precisely coordinated spatially and temporally according to biological and environmental cues [24]. In stem cell biology, these signals affect protein-protein interactions, which regulate the molecular processes of proliferation and differentiation. The systems biology approach of visualizing interacting proteins within a proteome presents in silico datasets as graphical a network representation at a global proteome scale and has become a significant tool in understanding the biological context in which proteins function and localize to promote complex cellular events.

The action of CKs have been previously shown to have an effect through primary interactions with thiomorpholine-carboxylate dehydrogenase and μ-crystallin proteins [9,10]. These initiating factors are likely responsible for potentiating the downstream crosstalk interaction between the MAP3K and Wnt/β-catenin pathways [25], which are vital in neural development. Complementary to pathway promotions, numerous correlative interacting protein targets and partners have statistical up regulation of and biologically relevant fold changes that support neurogenic differentiation, neurite migration as well as neuron and axon development. Furthermore, the reductive capacity of the chemicals also regulates oxidative stress and anti-inflammatory conditions. Whereby a substantial number of cellular damaging stress related proteins and cytokines, previously observed in BME treatment in Santos et al. [6], have been minimized in their expression. Therefore, consolidating the CKs gentle inductive capacity for neural differentiation. The investigation of the roles and interacting partners of up-regulated neural related proteins is important to comprehend the biological context of their expression.

Filamin-B was identified in both chemically treated ADSCs with a statistically significant up regulation in each differentiated cell. Filamin-B is an actin-binding protein that is highly expressed in the CNS, displaying roles in cellular migration and differentiation [26]. The relevance of Filamin-B in neurogenic differentiating ADSCs stems from its annotated role in neuroblast migration in the developing brain from the ventricular zone to the outer cortical plate [26]. The migratory roles are linked to the interaction between Filamin-B and its 70% homologous interacting partner Filamin-A, which promotes the development and maintenance of the cell’s bipolar shape [27]. The up regulation of Filamin-B in treated ADSCs is significant since the morphological shape of the treated cells appear elongated and bipolar prior to the development of neurite outgrowth (Figure 1G,H). An interesting binding partner was also identified within the statistically relevant proteins of both treatments. The partner; Ras-related C3 botulinum toxin substrate 1 (Rac1) is annotated with the biological process of nerve growth factor receptor signaling pathway and is a neural surface antigen. Rac1 is a pleiotropic regulator of a variety of cellular processes including the proliferation, differentiation, and neuronal maturation during embryonic and adult hippocampal development [28]. The assistance in axonal migration and dendritic development is directed via its signaling with a dense core of primary interacting proteins of the P21 protein (Cdc42/Rac) activated kinase family. This acts as a GTPase effector that links the action to the JNK/MAPK pathway regulating the spatial reorganization of the cytoskeleton for neural development and dendrite morphogenesis [29]. The identification of Rac1 in neuronal differentiating ADSCs indicates the morphological changes observed are maintained and directed through a number of control mechanisms that are widely seen in the development of in vivo neural tissue. Rac1 has the potential to be utilized as a novel marker for neurogenesis of ADSCs. The identification of Rac1 in neural differentiating ADSCs has the potential to be utilized as a novel marker for neurogenesis. This finding further supports the CK induction of ADSCs toward a neural phenotype.

Thioredoxin and Peroxiredoxin-1 were abundantly expressed in both chemical treated ADSCs. Each protein is found ubiquitously in mammalian cells [30] thus they would not be suitable as a neurospecific marker. Their function however is of great importance especially within the context of this study’s focus on chemical induction and role of low oxidative stress. All Peroxiredoxins contain a conserved cysteine residue which undergoes a cycle of peroxide-dependent oxidation and thiol-dependent reduction [31]. This is particularly relevant since it regulates the intracellular redox state, effectively protecting the cells from oxidative stress. This may occur, to a certain extent, based on the oxidative-stress response proteins seen up regulated with BME treatment in a previous paper [6]. A study by Simzar et al. [32] found that the overexpression of peroxiredoxin in PC12 neuronal cells in vitro increased the presence of reactive oxygen species, essentially creating an oxidative stress inducing environment, this does not seem to be the case in this study as the cell population is maintained at sufficiently high numbers. The activity of Peroxiredoxin-1 is regulated by thioredoxin, which reduces the cysteine in peroxiredoxin [33]. The regulatory mechanisms maintaining the levels of peroxiredoxin and thioredoxin levels are essential in preserving cells in distress. A system of controlled oxidative stress has been shown to increase neurogenesis and oligodendrogenesis in adult neural progenitor cells [34]. The importance of expression and regulation of these two molecules in sustaining cells for proliferation and guiding neurogenesis is essential; furthermore, it can be used as a marker of the level of stress experienced by neurogenic induced cells.

The network dynamics across both AECK and LK treated cells presented a range of shared and highly interactive neuronal-related proteins, and the identification of Neuroblast differentiation-associated protein (AHNAK) as a hub for many interactions supports the conclusion that a differentiation process is occurring. Furthermore, AHNAK binding partners annexin2 and S100-A10 were also identified with at least a two-fold increase over basal levels. The established GO classification for AHNAK is its involvement in nervous system development. It has also been recognized to have multiple roles in neuronal development dependent on its cellular localization and calcium concentrations which regulates actin cytoskeleton organization, cell membrane architecture and cell-cell junction formation [35,36,37,38,39]. Moreover, the non-membrane bound AHNAK promiscuously interacts with several reported partners such as protein kinase C and phospholipase C which are involved in the activation of inositol metabolism [40,41,42]. Protein kinase C and phospholipase C were both found to be statistically significantly up regulated in both the AECK and LK treated ADSCs. Inositol metabolism is vital in regulating the Wnt/β-catenin pathway for nerve guidance, serotonin modulation and the control of intracellular calcium concentration [40,42]. There is mounting evidence for the usefulness of AHNAK as a marker for neurogenesis especially in the co-expression and up regulation of neuronal development related binding partners. This is further evidence that the CKs are more suitable for inducing neurogenesis of ADSCs.

An associated multifunctional-actin binding protein uniquely identified in the LK treated ADSCs is Gelsolin. Gelsolin localization and expression has been determined to be highest in the CNS and PNS and it has been found to be involved in a number of growth promoting and neuroprotective functions mediated possibly through the Wnt/β-catenin pathway [43]. Its role in actin remodeling in the nervous system is calcium dependent, with the protein initiating actin polymerization or disassembly. Since the highest expression has been determined to be in the oligodendrocytes and Schwann cells myelin sheaths, Tanaka et al. [43] proposed the role of gelsolin to be involved in the maturation of the myelin sheath forming cells. The neuroprotective capability is attributed to its anti-oxidative and anti-apoptotic functions in high oxidative-stress induced environments [44]. Interestingly the down regulation or proteolytic cleavage of gelsolin has been linked to the development of Alzheimer’s disease [45]. The up regulation of Gelsolin in the CK treated ADSCs and its value as a neuronal marker is apparent, since the presence of a neuronally related cytoskeletal remodelling protein with neuroprotective capabilities is an appealing find in the array of up regulated proteins of neurogenic differentiating ADSCs. This is further evidence that the CK chemicals are better suited for inducing neurogenesis in ADSCs.

Spectrin alpha and beta chain has been widely studied and are known to be major cytoskeletal components in the brain and distributed in the cytoplasm of neural cells and is modularly downstream regulated by the Wnt signaling pathway [46]. There is now evidence that spectrin regulates the surface chemistry and morphology of neuronal cells and large modifications or degradation would produce major modifications to synapses [46]. Spectrin has also been implicated in the calcium regulated release of neurotransmitters between developing synapses [47]. The regulation of spectrin is derived from the calcium modulated calpain proteolytic enzyme [46]. The levels of calpain in both chemical treatments in this study are negligible. Here the presence of a heterodimer of the brain isoforms of spectrin alpha and beta chain is well noted in the prospect of utility as a neural marker for further studies.

Lastly Galectin-3 increase in expression has been detected in the LK treated ADSCs. Recent studies have shown that galectin-3 is expressed in a variety of neuronal tissues, especially glial cells in the CNS in which it directs oligodendrocyte differentiation to control myelin sheath formation [48]. Furthermore, the control of neuroblast migration in brain development was proposed by Comte et al. [49]. A function that is more suited to the development of neuronal like cells was found by Pesheva et al., in which galectin-3 was found to stimulate neural cell adhesion and moreover, neurite outgrowth in developing cells [50]. This also provides further evidence that the ADSCs are responding favorably to the CK treatment, expressing neuronal-related proteins known to be functionally and structurally useful.

### 3.2. Neurogenic Roles of Cytokines

Cytokines are pleotropic proteins that coordinate signaling across varied tissues and cell types including during neural development [51]. Cytokines display functional roles in a variety of developmental stages, acting as neurotrophic factors initiating the repair and regeneration of cells [51]. Furthermore, certain groups of cytokines, such as chemokines, regulate the directed growth and communication between radially migrating neuronal cells which give rise to mature neurons, glial, astrocytes and oligodendrocytes [51]. Due to their relatively low abundance and the dynamic range of a proteome, these molecules are extremely difficult to detect by MS and alternative detection methods, such as the Bioplex cytokine, chemokine, and growth factor assay system, allows the relative quantitation and comparison of 27 secreted cytokines. Hierarchical group clustering was carried out to identify the cytokines that responded with similar trends during the differentiation process and possible roles within the CK treated cells relative to neurogenesis.

The cytokine expression changes in the CK treated ADSCs closely support the proteomic data displaying a similar broad change in concentrations and the thus cellular phenotype in response to treatment. The intimate view of the cytokines is vital in understanding their corresponding roles in neural development and growth. The concentration change of molecules from group 1 (IL-1ra, IL-2, MIP-1b, RANTES and MIP-1a) was apparent with the out of trend large fluctuation of MIP-1b and RANTES in the LK treated ADSCs (See supplementary material for detailed grouping). MIP-1b is a pro-inflammatory protein which displayed a substantial increase at 20 h in LK, indicating a significant pro-inflammatory affect. Interestingly, the increase in MIP-1b is mirrored by RANTES with a proceeding concentration increase at the same time point. The secretion of MIP-1b has been noted in a number of neuronal cells [52]. The developmental organization of neuronal cells and CNS development have been linked to a synchronous regulation of MIP-1b alongside other chemokines [53]. Studies have shown that MIP-1b knockout mice soon die after birth due to brain formation abnormalities [53]. Thus, the role of MIP-1b in ADSCs has gained some interest especially with a number of co-regulations at the same time point in other cytokine groups. RANTES, also a pro-inflammatory cytokine, was found to initiate neuro-protective roles assisting in the survival of stressed hippocampal cell lines in the presence of a toxin [54]. Interestingly a study conducted into investigating the effect of certain pain management drugs in patients with HIV found that morphine treatment deregulated and decreased the expression of RANTES in the neuronal tissue. This progressive effect was implicated in the decrease in microglial cell migration due to the suppression of the chemotactic cytokine [55]. Furthermore, another study into the effect of HIV glycoprotein120 on neuronal tissue presented a number of up regulated secreted pro-inflammatory cytokines including RANTES which offered a neuroprotective role [56]. These responses all lead to the stimulation of the CX3CL1, CCL4 and CCL5 receptors of the above cytokines [53] which may also be regulated in the differentiating ADSCs.

Examining beyond group 1 with the consideration of the 20 h time point, a number of similar variations in trends were identified amongst several cytokines in other groups. The decrease of IL-15 is a significant find as its expression and regulation, through an indirect neuroprotective mechanism, in astroglial cells has been found to be linked to RANTES, MIP-1a, MIP-1b and GM-CSF [57]. Interestingly IL-15, IL-17 and GM-CSF in LK’s group 5 present a massive decrease in concentration per cell from the 5 to 20 h time point. Elevated concentrations of IL-15 in neural stem cell (NSC) cultures have proven to reduce maturation and neurite out growth in differentiating neurons but not affect proliferation [58]. Furthermore, IL-15-deficient mice exhibited defective JAK/STAT and ERK pathways which are key in the regulation of differentiation in NSCs. This substantiates the possible role for the decrease of IL-15 in LK treated ADSCs which presented greater morphological differentiation than the AECK treated cells. The down regulation of IL-17 is also considered a beneficial decrease for cytokines in brain tissue, since elevated levels are usually present in a number of traumatic brain injuries or infarcts in which a large proportion of cells are damaged [59].

The cytokine (Bioplex) data complement the proteomic findings that the AECK and LK treatments of ADSCs initiate the differentiation toward a neural lineage and potentially neurons, evidenced by the proteomic and microscopic analyses. The results also indicate the ADSCs favor the treatment with LK over AECK for neuronal-like differentiation within the 24 h treatment time. Notwithstanding the AECK did produce a slightly higher cell population at the end of the differentiation time and similar neurogenic-related proteins were identified. The cytokine data also are consistent with a lack of cytotoxic stress of the cells over the period examined, thus removing cytotoxicity as a cause for the neuronal-like morphological changes [7].

## 4. Materials and Methods

### 4.1. Cell Culture

#### 4.1.1. Human Adipose Derived Stem Cells Harvest and Cell Culture

The procedures of adult ADSCs isolation and expansion were used from Santos et al., 2017 [6] in accordance with guidelines and regulations under Macquarie University Human Research Ethics Committee approval (MQ-HREC Ref #: 5201100385, 8 March 2011). All donor participants volunteered through informed consent for lipoaspirate donation as per ethics guidelines and were de-identified for research purposes. Generally, ADSCs were maintained in T175 flask (Nunc, ThermoScientific, Carlsbad, CA, USA) in 15 mL D-MEM Glutmax/F12 (Gibco, Life Technologies, Carlsbad, CA, USA) with 10% Foetal Bovine Serum (FBS, Gibco, Life Technologies, Carlsbad, CA, USA) and 1% Antibiotics/Antimycotics (ABAM, Gibco, Life Technologies, Carlsbad, CA, USA) incubated at 37 °C at 5% ADSCs were passaged 3–5 post isolation times by stripping cells with TrypLE Express (12604 Gibco, Life Technologies, Roskilde, Denmark) before being utilized in differentiation experiments.

#### 4.1.2. Chemical Induction for Differentiation

Sub-confluent ADSCs were washed twice in pre-warmed sterile D-MEM/F12 (Gibco, Life Technologies, Carlsbad, CA, USA). The cells were then cultured for a further 24 h in a serum-free pre-induction medium consisting of D-MEM/F12 (Invitrogen), ABAM (Gibco, Life Technologies, Carlsbad, CA, USA) and 10% of the final concentration of the added Ketamine. The media was then replaced after 24 h with the neuronal inducing media consisting of D-MEM/F12, ABAM and the final optimized concentrations of 0.5 mM AECK, or 0.6 mM LK, or 0.3 mM LKEE.

#### 4.1.3. Glioblastoma Cell Culture

The GBC line was cultured in neurobasal media supplemented with B27 and 0.5 mM Glutamine (Gibco, Life Technologies, Carlsbad, CA, USA). The cells were grown to 90% confluence prior to passaging or harvesting for proteomics.

### 4.2. Microscopy

#### Cell Counts

In vitro cell counts were carried out utilizing a procedure described in Santos et al., 2017 [6] to determine the approximate colony forming units per square millimeter. The total cell number data was also utilized in the Bioplex analysis to determine the amount of cytokines secreted per cell. This was calculated by multiplying the concentration by the total volume of the flask and dividing by the total cell number at the respective time point.

### 4.3. Protein Extraction

Harvesting cells for proteomic analysis by LC-MS/MS or iTRAQ and Western blot (see Supplementary Material) were completed at the desired end time point as per Santos et al., 2017 [6].

### 4.4. iTRAQ

iTRAQ labelling and mass spectrometry were followed as per [6] with the following modifications; after cell lysis and protein extraction, there were a total of 4 samples for iTRAQ labelling (1—ADSCs, 2—AECK treated hADSC, 3—LK treated hADSC and 4—Glioblastoma control (GBCs)). AECK and LK data sets checked for normalized data distribution and volcano plots were generated using DanteR software [59]. Post hoc interaction network analysis was performed on Cytoscape (version 3.5.1, Cytoscape Consortium, Seattle, WA, USA) [11].

### 4.5. Bioplex

Bioplex analysis was performed as per Santos et al., [6] with 500 µL aliquots collected differentiation timepoints at 0, 0.5, 1, 3, 5, 20 and 24 h Assay was performed with Bioplex human 27-plex (M50-0KCAF0Y Bio-Rad Laboratories, Hercules, CA, USA). Data analysis was completed in DanteR software (DanteR version 1.0.0.10. R version 2.12.0 The R Foundation for Statistical Computing, Auckland, New Zealand) [59].

## 5. Conclusions

This study aimed to investigate the extent of proteomic change in ADSCs treated with two different CKs compounds LK and AECK for the purpose of a directed neurogenic induction differentiation. The extent of differentiation was investigated by the changes in the proteome occurring during the process. The treatments indicated that the ADSCs responded favorably to the neurogenic induction media by presenting a number of morphological cues previously observed [4,6] and a higher cell population post induction compared to previous studies with BME [6]. Furthermore, a variety of proteins were identified by mass spectrometric analyses a number of neurogenic and stress related proteins have been up-regulated, most of which are noted in the literature to have positive effects for neurogenic differentiation. A number of the statistically significant proteins were explored, investigating their known function in developing neurons and their associated role within the treated cells. Complementing the iTRAQ quantitative proteomic data, the Bioplex system allowed the investigation of a closed cohort of cytokines and interleukins allowing trends to be examined, allowing a closer look at the smaller and lower copy number proteins that have numerous profound affects in the immunogenicity and stress response of cells.

Here we have shown that the treatment of ADSCs with the CK compounds, AECK and LK, have the potential to induce ADSCs toward a neurogenic phenotype, producing similar morphological traits established in previous studies but with the added benefit of being seemingly non-toxic at the utilized concentrations as indicated by cell counts. The supporting evidence of the expression of a wide range of neural-related proteins which have not been previously utilized as neural markers but are known to play an integral role in the neuronal maturation and development of the CNS, further infers the CK’s differentiation function. Thus, the novel application of CK’s to produce a neurogenic cell population within 24 h of induction holds potential for further applications for neuroregeneration and in studies in the transdifferentiation of ADSCs. Future directions will explore neuronal maturation and neuronal activity by action potential measurement.

## Figures and Tables

**Table 1:** Number of proteins and peptides identified by ProteinPilot after iTRAQ LC/MS/MS analysis of human adipose derived stem cells (hADSCs), *S*-aminoethyl-l-cysteine ketamine (AECK) differentiated, LK differentiated and Glioblastoma cells. Confidence cutoffs are the proteins with significant change detected between labels.

Confidence Cutoff	Proteins Detected	Proteins before Grouping	Distinct Peptides	Spectra Identified	% Total Spectra
>2.0 (99)	2108	2774	35,891	176,734	67.3
>1.3 (95)	2430	3204	36,993	178,574	68.0
>0.47 (66)	2741	5246	38,381	180,467	68.8
Cutoff applied: >0.05 (10%)	3491	15,271	41,011	184,078	70.1

**Table 2:** Summary of biologically and statistically significant up-regulated neural-related proteins identified in iTRAQ of hADSCs treated with AECK.

AECK Neural	Accession	Name	GO	Biological Process	Peptides (95%)	Fold Change	*p*-Value
	O75369	Filamin-B	GO:0030154	cell differentiation	129	1.230453968	2.44 × 10^−15^
	P00367	Glutamate dehydrogenase 1, mitochondrial	GO:0006537	glutamate biosynthetic process	12	1.339429975	0.01273619
	P09429	High mobility group protein B1	GO:0031175	neuron projection development	16	1.15634203	0.03726548
	P10599	Thioredoxin	GO:0008283	cell proliferation	7	1.57030201	0.04286075
	P15144	Aminopeptidase N	GO:0030154	cell differentiation	32	1.223500967	1.10 × 10^−5^
	P15559	NAD(P)H dehydrogenase (quinone) 1	GO:0007271	synaptic transmission, cholinergic	9	1.816967964	0.002394901
	Q00610	Clathrin heavy chain 1	GO:0048011	nerve growth factor receptor signalling pathway	69	1.205008984	7.17 × 10^−7^
	Q01082	Spectrin beta chain, brain 1	GO:0007411	axon guidance	54	1.284289956	3.63 × 10^−10^
	Q06830	Peroxiredoxin-1	GO:0008283	cell proliferation	25	1.434638977	0.008896183
	Q09666	Transforming protein RhoA	GO:0007399	nervous system development	16	1.167513967	0.019935589
	Q09666	Neuroblast differentiation-associated protein AHNAK	GO:0007399	nervous system development	285	1.527696013	1.40 × 10^−45^
	Q13813	Spectrin alpha chain, brain	GO:0007411	axon guidance	71	1.29076004	6.27 × 10^−14^
	P63000	Ras-related C3 botulinum toxin substrate 1	GO:0048011	nerve growth factor receptor signaling pathway	7	1.664183	0.006588
	Q9P0L0	Vesicle-associated membrane protein-associated protein A	GO:0031175	neuron projection development	10	1.441416979	0.006864889

**Table 3:** Summary of biologically and statistically significant up-regulated neural-related proteins identified in iTRAQ of hADSCs treated with LK.

LK Neural	Accession	Name	GO	Biological Process	Peptides (95%)	Fold Change	*p*-Value
	O75369	Filamin-B	GO:0030154	cell differentiation	129	1.372761965	0.0149013
	P06396	Gelsolin	GO:0060271	cilium morphogenesis	23	1.32772994	0.001008915
	P10599	Thioredoxin	GO:0008283	cell proliferation	4	1.76563704	0.02659229
	P11142	Heat shock cognate 71 kDa protein	GO:0007269	neurotransmitter secretion	73	1.381860971	0.00224432
	P11413	Glucose-6-phosphate 1-dehydrogenase	GO:0001816	cytokine production	23	1.242061019	0.00224432
	P15144	Aminopeptidase N	GO:0030154	cell differentiation	32	1.44699502	0.019935589
	P17931	Galectin-3	GO:0030154	cell differentiation	7	1.757151961	0.006587825
	Q01082	Spectrin beta chain, brain 1	GO:0007411	axon guidance	54	1.506716013	6.27 × 10^−14^
	Q09666	Neuroblast differentiation-associated protein AHNAK	GO:0007399	nervous system development	285	1.659075022	2.00 × 10^−25^
	Q13813	Spectrin alpha chain, brain	GO:0007411	axon guidance	71	1.395959973	6.27 × 10^−14^
	Q92974	Rho guanine nucleotide exchange factor 2	GO:0048011	nerve growth factor receptor signaling pathway	9	1.163854957	0.033079
	P63000	Ras-related C3 botulinum toxin substrate 1	GO:0048011	nerve growth factor receptor signaling pathway	7	1.616315960	0.006153737
	Q9P0L0	Vesicle-associated membrane protein-associated protein A	GO:0031175	neuron projection development	10	1.531931043	0.039667

**Table 4:** Summary of significant up-regulated stress-related proteins identified in iTRAQ of hADSCs treated with AECK.

AECK Stress	Accession	Name	GO	Biological Process	Peptides (95%)	Fold Change	*p*-Value
	P01892	HLA class I histocompatibility antigen, A-2 alpha chain	GO:0060333	interferon-gamma-mediated signaling pathway	4	1.4692	0.01600371
	P04083	Annexin A1	GO:0006954	inflammatory response	45	1.3066	0.00108737
	P04264	Keratin, type II cytoskeletal 1	GO:0006979	response to oxidative stress	13	2.0287	0.00100892
	P06396	Gelsolin	GO:0006921	cellular component disassembly involved in apoptosis	25	1.3732	2.05 × 10^−13^
	P09429	High mobility group protein B1	GO:0002437	inflammatory response to antigenic stimulus positive regulation of apoptosis	16	1.1563	0.00754318
	P11413	Glucose-6-phosphate 1-dehydrogenase	GO:0034599	cellular response to oxidative stress	23	1.2994	0.00129321
	P15121	Aldose reductase	GO:0006950	response to stress	9	1.3973	0.00414161
	P16070	CD44 antigen	GO:0060333	interferon-gamma-mediated signaling pathway	15	1.3484	8.89 × 10^−5^
	P30044	Peroxiredoxin-5, mitochondrial	GO:0034614	cellular response to reactive oxygen species inflammatory response	9	1.3299	0.00063648
	P35611	Alpha-adducin	GO:0006921	cellular component disassembly involved in apoptosis	12	1.5308	4.06 × 10^−12^
	P51572	B-cell receptor-associated protein 31	GO:0006921	cellular component disassembly involved in apoptosis	7	1.4834	0.00252419
	P61586	Transforming protein RhoA	GO:0050772	positive regulation of axonogenesis	16	1.1675	0.00013793
	P63000	Ras-related C3 botulinum toxin substrate 1	GO:0008624	induction of apoptosis by extracellular signals	7	1.6642	3.48 × 10^−6^
	P63000	Ras-related C3 botulinum toxin substrate 1	GO:0006954	inflammatory response	7	1.6642	0.00016355
	P63241	Eukaryotic translation initiation factor 5A-1	GO:0006917	induction of apoptosis	20	1.4873	6.36 × 10^−12^
	Q02952	A-kinase anchor protein 12	GO:0030819	positive regulation of cAMP biosynthetic process	17	1.3261	0.0067851
	Q03135	Caveolin-1	GO:0009267	cellular response to starvation; inactivation of MAPK activity; positive regulation of calcium ion transport into cytosol; positive regulation of canonical Wnt receptor signaling pathway; response to hypoxia	7	3.1097	0.02121297
	Q13813	Spectrin alpha chain	GO:0006921	cellular component disassembly involved in apoptosis	71	1.2908	0.00131438
	Q15149	Plectin	GO:0006921	cellular component disassembly involved in apoptosis	174	1.3019	2.20 × 10^−9^
	Q9NR28	Diablo homolog, mitochondrial	GO:0008625	induction of apoptosis via death domain receptors	6	1.2655	2.20 × 10^−9^

**Table 5:** Summary of significant up-regulated stress-related proteins identified in iTRAQ of hADSCs treated with LK.

LK Stress	Accession	Name	GO	Biological Process	Peptides (95%)	Fold Change	*p*-Value
	P02545	Prelamin-A/C	GO:0006921	cellular component disassembly involved in apoptosis	63	1.4487	3.04 × 10^−9^
	P06396	Gelsolin	GO:0006921	cellular component disassembly involved in apoptosis	23	1.3277	0.00031779
	P08670	Vimentin	GO:0006921	cellular component disassembly involved in apoptosis	199	1.2634	7.44 × 10^−5^
	P11413	Glucose-6-phosphate 1-dehydrogenase	GO:0034599	cellular response to oxidative stress	23	1.2421	0.00100042
	P35611	Alpha-adducin	GO:0006921	cellular component disassembly involved in apoptosis	12	1.5308	0.02401391
	P36776	Lon protease homolog, mitochondrial	GO:0034599	cellular response to oxidative stress	17	1.4223	0.0050889
	P51572	B-cell receptor-associated protein 31	GO:0006921	cellular component disassembly involved in apoptosis	7	1.5205	0.00379271
	Q03135	Caveolin-1	GO:0009267	cellular response to starvation; inactivation of MAPK activity	7	2.5188	0.00436605
	Q13813	Spectrin alpha chain, brain	GO:0006921	cellular component disassembly involved in apoptosis	71	1.396	9.24 × 10^−15^
	Q15149	Plectin	GO:0006921	cellular component disassembly involved in apoptosis	174	1.569	2.06 × 10^−39^

## Supplementary Materials

Supplementary materials can be found at http://www.mdpi.com/1422-0067/20/3/523/s1.

## Abbreviations

AECK	*S*-aminoethyl-l-cysteine ketamine
BHA	butylated hydroxyanisole
BME	beta mercaptoethanol
CK	cyclic ketamine
DMSO	dimethylsulfoxide
hADSCs	human adipose derived stem cells
iTRAQ	Isobaric tag for relative and absolute quantitation
LK	lanthionine ketamine
LKEE	lanthionine ketamine ethyl ester
MSC	mesenchymal stromal/stem cells
MSMS	tandem mass spectrometry
PBS	phosphate buffered saline
