# Correction: Santos, J., *et al.* Proteomic Analysis of Cyclic Ketamine Compounds Ability to Induce Neural Differentiation in Human Adult Mesenchymal Stem Cells. *International Journal of Molecular Sciences* 2019, *20*, 523

**DOI:** 10.3390/ijms20143542

**Published:** 2019-07-19

**Authors:** Jerran Santos, Bruce Kenneth Milthorpe, Matthew Paul Padula

**Affiliations:** 1 Advanced Tissue Regeneration & Drug Delivery Group, School of Life Sciences, University of Technology Sydney, P.O. Box 123 Broadway, Ultimo 2007, Australia; Bruce.Milthorpe@uts.edu.au; 2 Proteomics Core Facility and School of Life Sciences, Faculty of Science, University of Technology Sydney, P.O. Box 123 Broadway, Ultimo 2007, Australia; Matthew.Padula@uts.edu.au; 3 CIRIMAT, Paul Sabatier, University of Toulouse 3 (INPT), 118 Route de Narbonne, 31062 Toulouse, France

The authors wish to make the following corrections to this paper [1] in the Acknowledgements:

Dr. Hallen has requested that his name be removed from the Acknowledgments. The authors wish to apologize to Dr. Hallen for including his name in the Acknowledgements. The corrected Acknowledgements section should be:

We would like to acknowledge UTS and MQU for internal project funding. Special thanks to Prof. Jim Piper for R&D internal funding for patent development, Bill Russell and Tim Dably for Patent Management, and the Australian Proteome Analysis facility (APAF), Macquarie University, Thiri Zaw, for technical support.

The authors would like to apologize for any inconvenience caused to the readers by these changes.
