# Supplementary material for: Proteomic Analysis of Cyclic Ketamine Compounds Ability to Induce Neural Differentiation in Human Adult Mesenchymal Stem Cells
Source: Int J Mol Sci. 2019 Jan 26;20(3):523. doi: 10.3390/ijms20030523 (PMC6387408; doi:10.3390/ijms20030523)
Supplement: Supplementary file 1 [file ijms-20-00523-s001.zip › supplementary/Supplementary Material.docx]

**Supplementary Material**

**Two -Dimensional SDS-PAGE (2-DE) experimental procedure**

All protein extraction was performed in the procedure outlined Santos et al., [21] and isoelectric focusing performed using a modified method from Jobbins *et al* [1]. The whole cell lysates protein extract was separated in the first dimension on an IPG strip (11 cm, pH 4−7 or pH 3−10, BioRad) for at least 100kVhours. The focused IPG strip was equilibrated in 2% (w/v) SDS, 6 M Urea, 250 mM Tris-HCl (pH 8.5) and then separated in the second dimension on a 4−12% Bis-Tris gel using the MES buffer system at 160 V. Following separation and fixation, gels were visualized by staining with either Flamingo Fluorescent Gel Stain (BioRad, Australia) or Coomassie Blue G-250 [1]. A differential display analysis utilising PDQuest (BioRad) software was utilised to identify difference in 2D-gel profiles.

**Protein band excision and extraction for in-gel trypsin digestion and LC-MS/MS experimental procedure**

Gel spots (2D) stained with Coomassie Blue G250 were excised and destained with 50% acetonitrile (ACN) in 50 mM ammonium bicarbonate until the blue colour was no longer visible. Gel pieces were then dehydrated with 100% ACN for 5 minutes before removing ACN. Reduction and alkylation was then carried out by incubating the gel pieces with 100 μL 5 mM TBP and 20 mM acrylamide in 100 mM Am for 90 minutes. The solution was discarded and gel pieces washed first with 100 μL 100 mM ammonium bicarbonate and then twice with 50% ACN in 50 mM ammonium bicarbonate followed by dehydration with 100 μL ACN. Once the gel pieces had shrunk and turned noticeably white, Trypsin Gold, MS grade [Promega USA] in 100 mM ammonium bicarbonate (12.5 ng/μL) was added and left to incubate for 30 minutes at 4^0^C. The sample was spun briefly and more 100 mM NH4HCO3 added to cover the pieces before digestion overnight at 37^0^C. Peptides were extracted by sonicating briefly and transferring the digestion solution into a new tube. To the gel pieces 50% ACN and 2% formic acid was added and incubated for 20 minutes before sonicating again and removing the liquid to combine with initial digest solution. This last step was repeated before the digestion solution was concentrated to approximately 15 μL using a VacufugeTM Concentrator 5301 [Eppendorf Germany] and then centrifuged at 14,000 xg for 10 minutes. The solution was then transferred to an autosampler vial for LC-MS/MS analysis.

Once samples were prepared for analysis, all subsequent MS operations were performed by Dr Matt Padula from the Proteomics Core Facility at UTS using the Tempo/QSTAR Elite system and standardized automated methods. Samples were placed onto the autosampler of a TEMPO^TM^ nanoLC system [Eksigent USA] and loaded at a rate of 20 μL per minute onto a Michrom reverse phase trapping cartridge before eluting onto a 75 μmID X 150 mm PicoFrit column [New Objective, USA] packed with Magic C18AQ chromatography resin [Bruker-Michrom, USA]. Peptides were separated using an increasing gradient of ACN at 300 nL/minute and ionised at 2300 V by the Microlonspray II head into the source for the QSTAR EliteTM Quadrupole TOF MS [Applied Biosystems/MDS Sciex]. The QSTAR performs IDA to analyse ions transmitted through the first quadrupole to the TOF analyser. If a multiply charged ion (2-5+) was detected at greater than 30 counts per scan, the ion was selected and transmitted to the second quadrupole collision cell to be fragmented and the fragment masses measured by the TOF analyser.

The MS/MS data files produced by the QSTAR were searched using Mascot Daemon (version 2.3.02, provided by the Walter and Elisa Hall Institute, Parkville, Vic. Perkins, D.N. 1999) and searched against the LudwigNR database (comprised of the UniProt, plasmoDB and Ensembl databases (vQ212. 19375804 sequences, 6797271065 residues) with the following parameter settings: Fixed modifications: none; Variable modifications: propionamide, oxidised methionine, deamidated asparagine and glutamine; Enzyme: semitrypsin; Number of allowed missed cleavages: 3; Peptide mass tolerance: 100 ppm; MS/MS mass tolerance: 0.2 Da; Charge state: 2+ and 3+. The results of the search were then filtered by including only protein hits with at least one unique peptide and excluding proteins identified by a single peptide hit with a p-value > 0.05.

**Supplementary Results**

Prior to the treatment of human ADSCs, varying concentrations of LK, AECK and their ethyl ester derivatives were trialled against rat ADSCs to investigate the extent of a morphological response that may indicate neurogenic-like growth. The optimal concentrations tested which elicited a cellular response in which the produced cell’s morphology resembled neuronal like outgrowth within 24 hours were 0.5 mM AECK, 0.6 mM LK and 0.3 mM LKEE. In terms of morphological appearance, the AECK and LK produced the most cells with neuronal-like appearance which presented neurite-like out growth (Figure 1 A and B). The ethyl ester produced fewer morphologically differentiated cells that presented neurite outgrowth at the final time point however it did produce a large number of spheroid, semi-detached cells (Figure 1 C). The downfall of the ethyl ester derivatives was a low cell population at the final time point which was thought to be due to the increased membrane permeability which may have induced a toxic intracellular environment evidenced by the higher proportion of lysed cells. Concentrations lower than the above stated did not yield a response at the given time point.


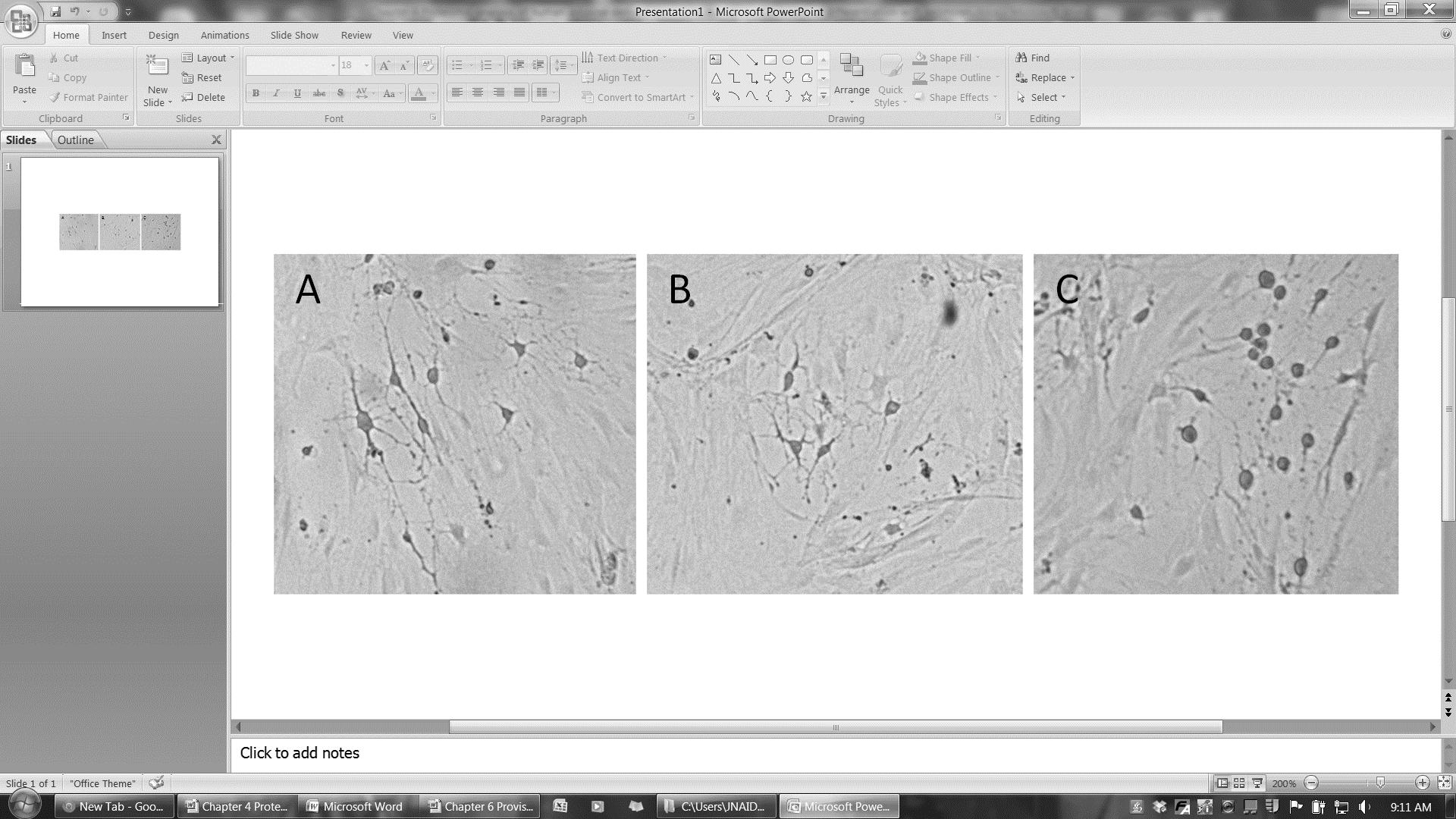


**Figure 1.** Rat ADSCs treated with AECK (A), LK (B) and LKEE (C) for 24 hours. Cells appear morphologically distinct with neurite-like extensions produced from cells compared to surrounding non-differentiated ADSCs.

The treated cells were also investigated by 2D SDS-PAGE to establish any broad proteome differences between the basal ADSCs and the CK treated (Figure 2 A-D). While the profiles appear to retain a number of similarities, the outstanding differences appear in expression levels variances in a number of the marked areas. These variable protein spots were excised and the protein preparations were tryptically digested for LC-MS/MS analysis. This identified a wide variety of proteins including a number of neurogenic related proteins unique to the CK treated rADSCs. Based on the higher yields of cells presenting morphological cues as well as a number of neuronal markers, the AECK and LK molecules were selected for testing on human ADSCs to be analysed via iTRAQ and Bioplex.


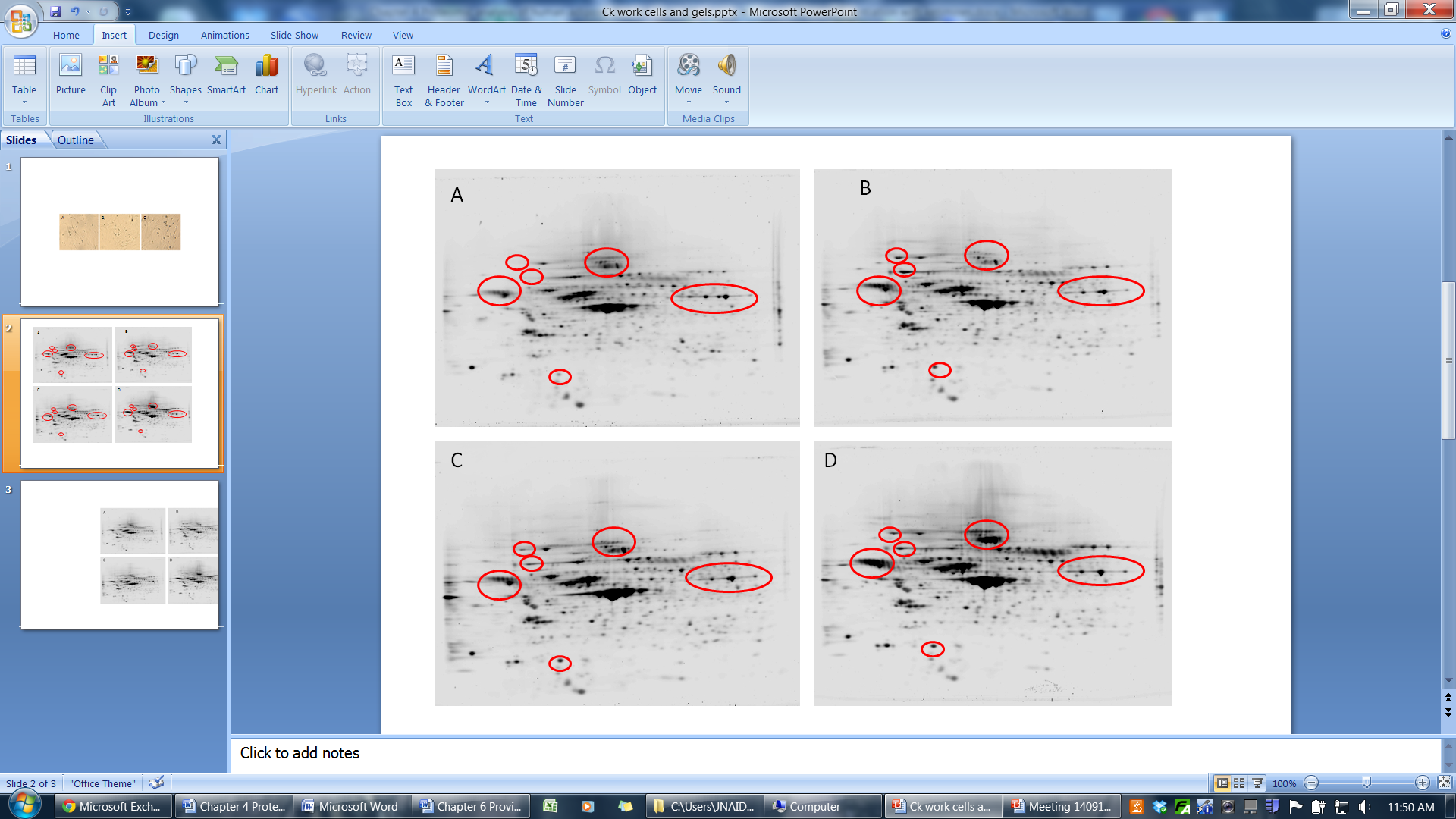


**Figure 2.** 2D-PAGE of rat ADSCs treated with AECK (A), LK (B) and LKEE (C) with basal rADSCs (D) for proteome change comparisons which some examples are generally marked in red circles.


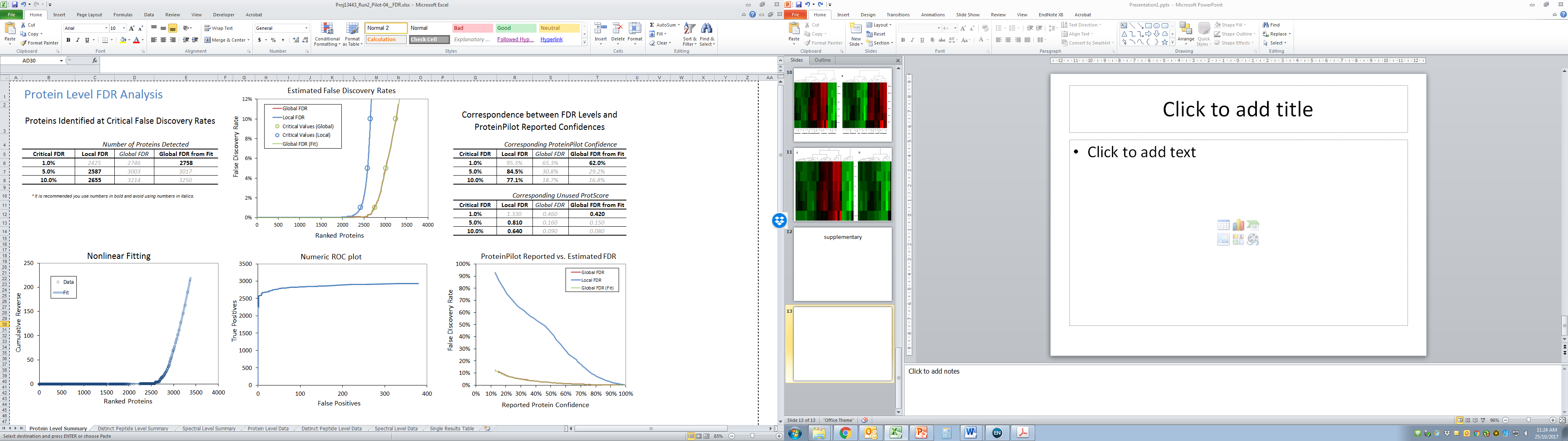


A


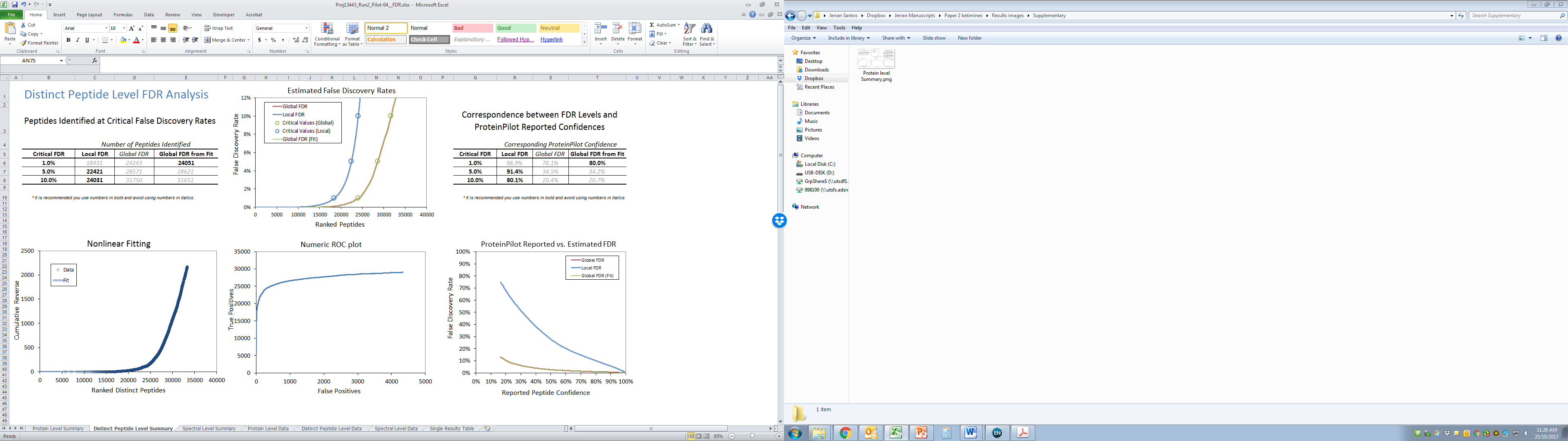


B


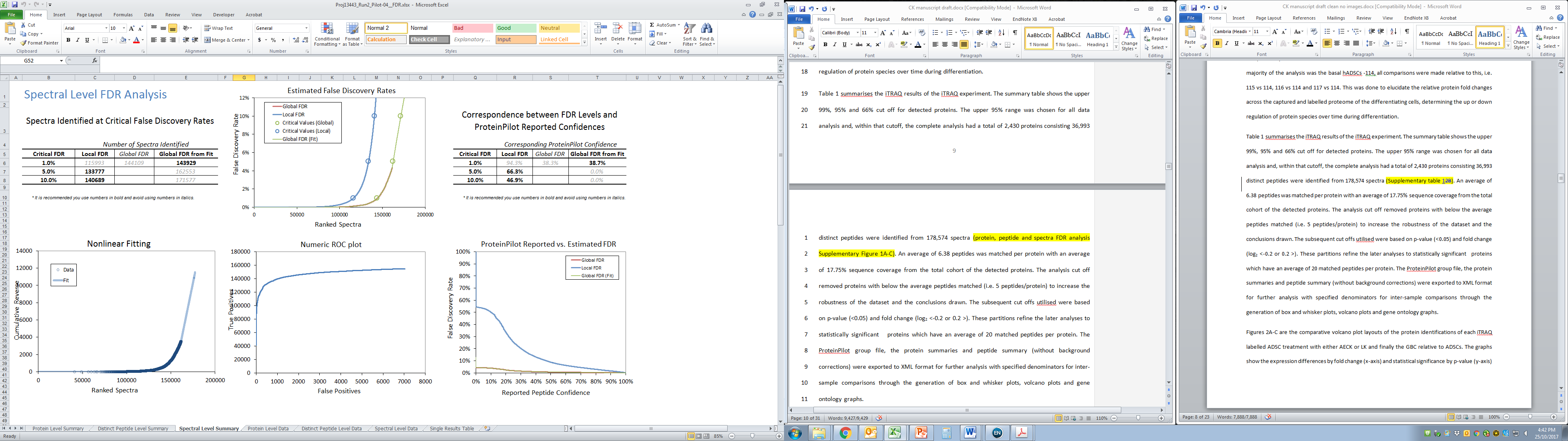


C

**Figure 3.** iTRAQ run (A) Protein Level FDR Analysis (B) Peptide Level FDR Analysis (C) Spectra Level FDR analysis.

**Bioplex Normalisation See Table 3A and 3B**

**Bioplex Trend Graphs**

Group 1 (Figure 4) comprises of IL-1ra, IL-2, MIP-1a, MIP-1b and Rantes which generally follows the trend where the highest concentration occurs in the basal cells, the concentration then decreases by approximately 50% post induction and remains at that level for the remainder of the time points. The variation of the group trend is identified between the comparison of the AECK and LK MIP-1b and Rantes levels. The MIP-1b in the LK treatment increases above the basal ADSC concentration at the 20 hr time point. Similarly at the same time point in the LK treatment, the Rantes concentration increases breaking trend with the rest of the cohort relative to the AECK samples. The trends for this group of cytokines is vastly different from BME treated ADSCs. The main difference is the types of cytokines present, which the BME treated cells lack the MIP-1a and MIP-1b however has Eotaxin.


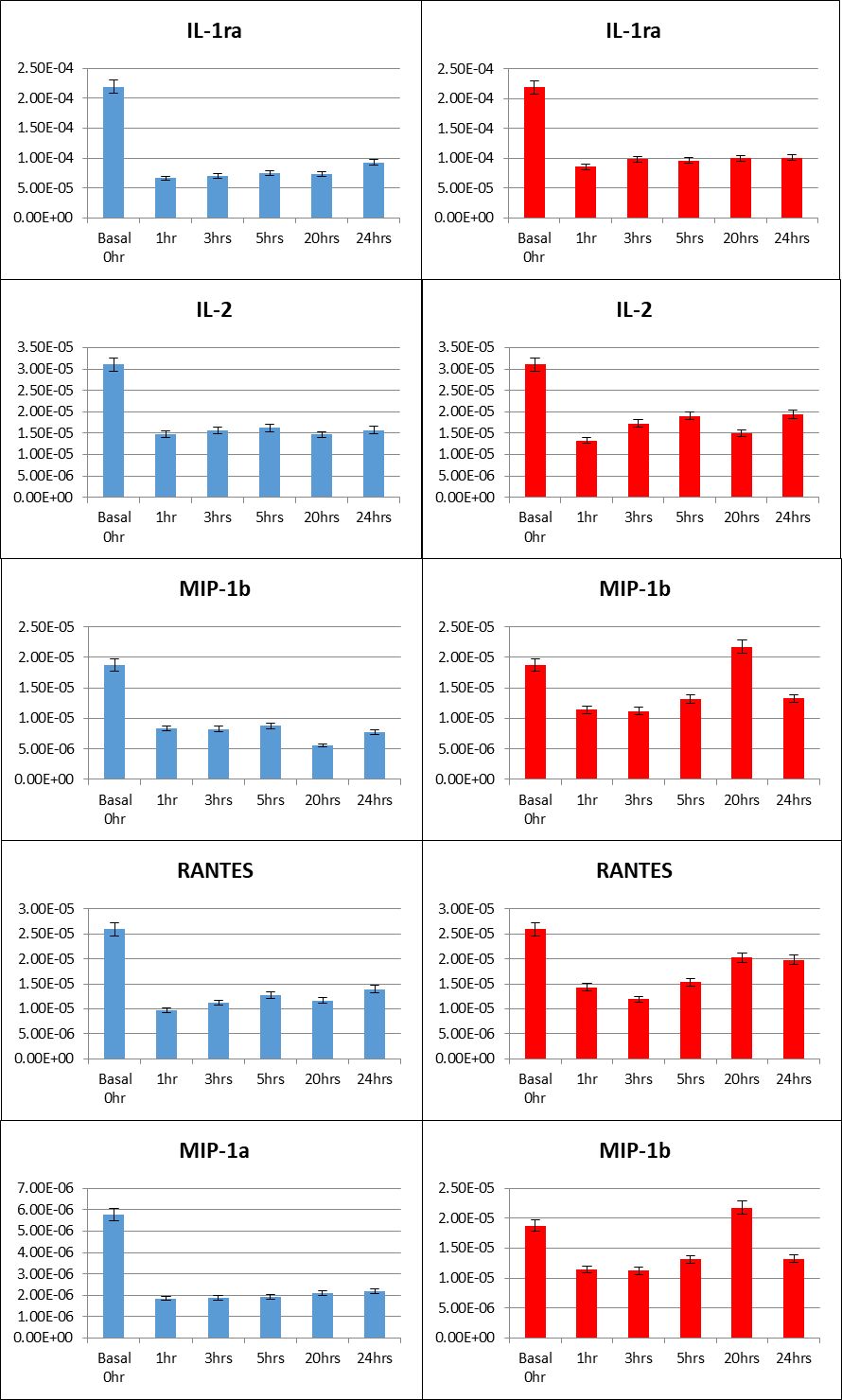


Figure 4. Group 1 Bioplex trend related secreted cytokines IL-1ra, IL-2, MIP-1b, Rantes and MIP-1 over a temporal differentiation normalised as pg/cell (AECK - Blue, LK – Red).

Group 2 (TNF-α, IL-10, IL-7, IL-4, IL-12 and IL-13) (Figure 5) presents a similar trend to group 1 wherein the highest concentration is present in the basal untreated ADSCs which then decreases to less than a quarter of the basal levels with a minor recovery across all cytokines after the 20 hr time point.


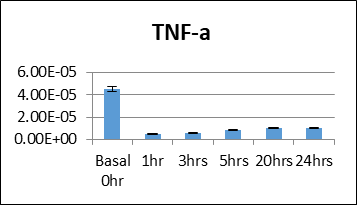

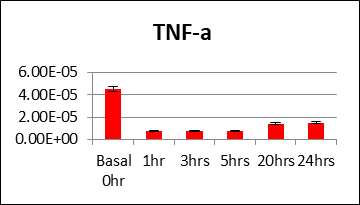

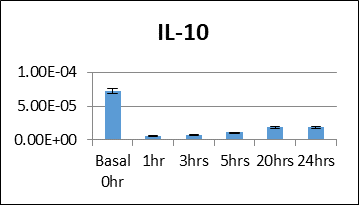

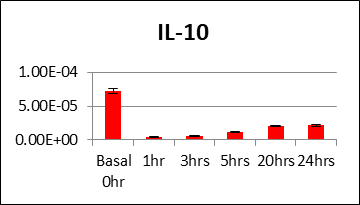

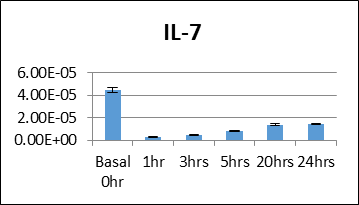

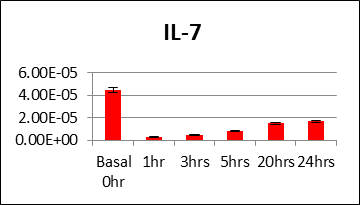

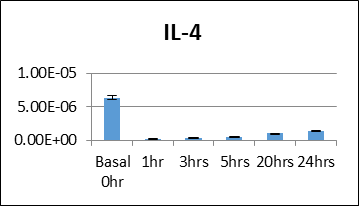

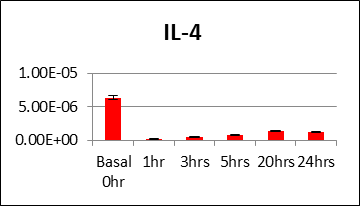

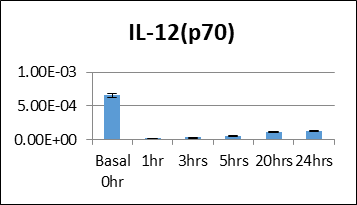

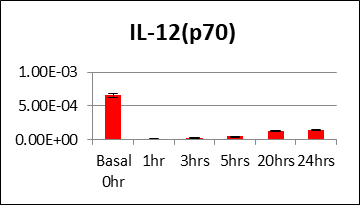

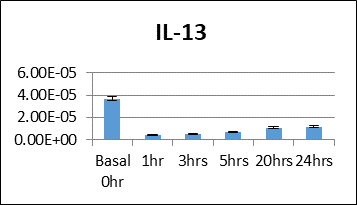

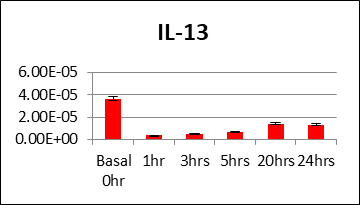


Figure 5. Group 2 Bioplex trend related secreted cytokines IL-7, IL-4, TNFα, IL-10, IL-12 and IL-13 over a temporal differentiation normalised as pg/cell (AECK - Blue, LK - Red).

Group 3 (Figure 6), comprised of PDGF-bb, IFN-γ and MCP-1 also shares the high basal concentrations in common with group 1 and 2 where a substantial decrease within the first hour is followed by minor recover for every time point thereafter. The differences in cytokine concentration between the AECK and LK samples are infinitisimal within this group . An interesting trend variation within this group occurs for both the AECK and LK MCP-1 which assumes an approximately 50% concentration recovery by the 20^th^ hr time point which increases again at the 24^th^ hour time point.


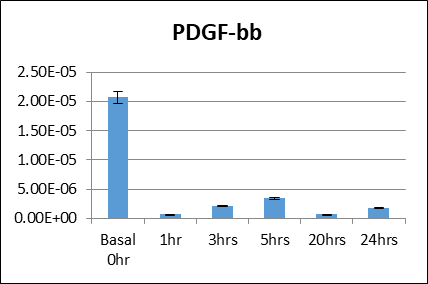

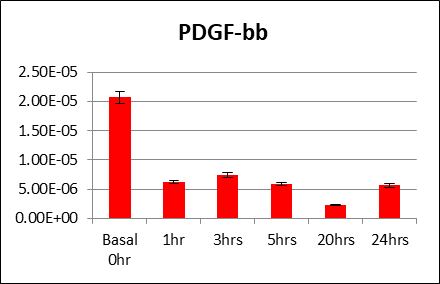

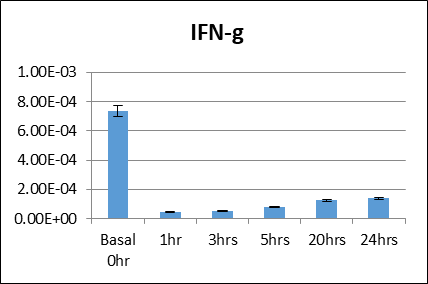

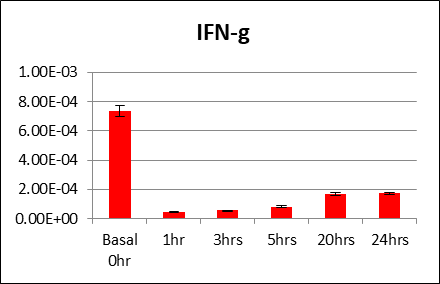

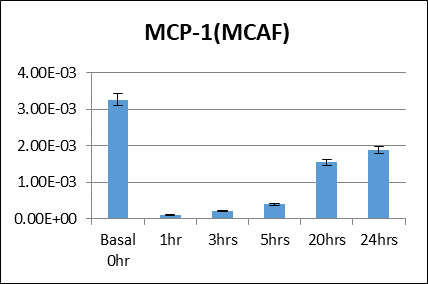

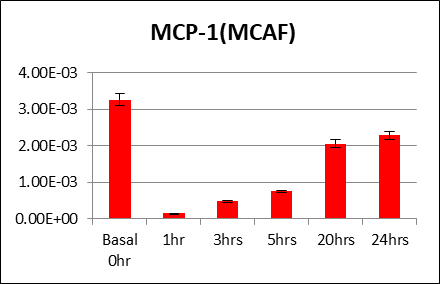


Figure 6. Group 3 Bioplex trend related secreted cytokines PDGF-bb, IFNγ and MCP-1 over a temporal differentiation normalised as pg/cell (AECK - Blue, LK - Red).

Group 4 (figure 7) consisting of the three molecules, Eotaxin, IL-5 and IL-9, has a simple trend in which the large decrease in basal levels after 1 hour post induction is observed again as per previous groups. The unique feature of this group is the concentration recovery for every time point thereafter to at least 50% of the basal ADSC levels is seen for IL-5 and IL-9. Eotaxin however returns to basal levels for the AECK treatment and surpasses basal level in the LK treatment.


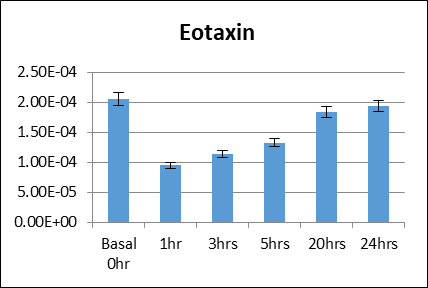

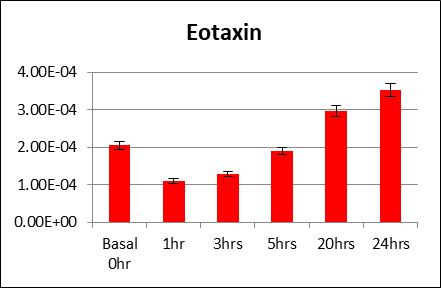

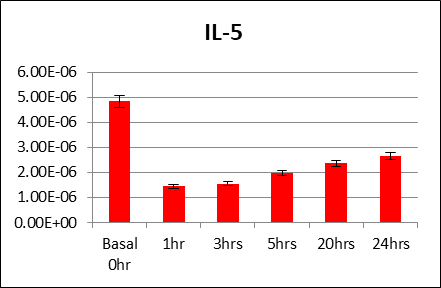

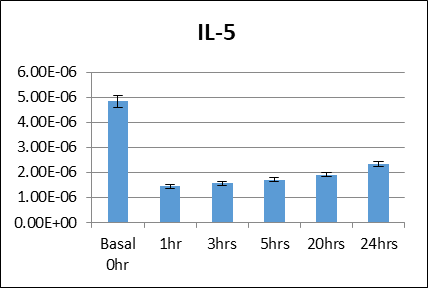

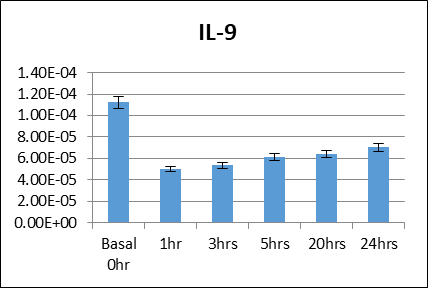

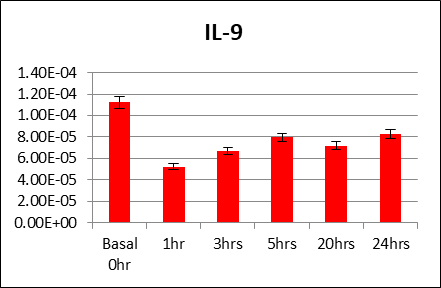


**Figure 7.** Group 4 Bioplex trend related secreted cytokines Eotaxin, IL-5 and IL-9 over a temporal differentiation normalised as pg/cell (AECK - Blue, LK - Red).

Group 5’s (figure 8) molecules present the greatest variation between AECK and LK treatments of ADSCs. The trends within this group are not as consistent as previous groups; the main factor clustering these molecules together is the overall relative similarity of their concentrations. The difference between the AECK and LK IL-15 is noteworthy, where each time point presents a variation in the concentration compared to the other. The most noteworthy variances are present at 1 hour and 20 hours post induction where the LK’s IL-15 concentration is significantly lower than the AECKs. Conversely the concentration of IL-17 and GM-CSF in the LK sample is higher at all time points compared to the AECK sample.


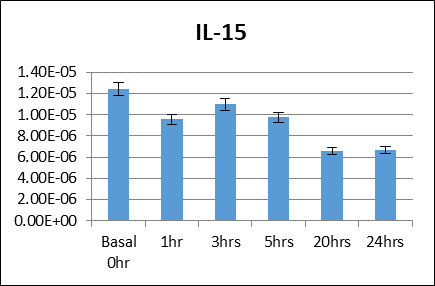

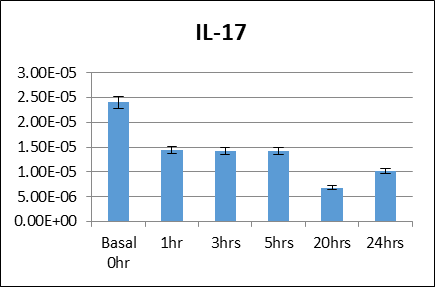

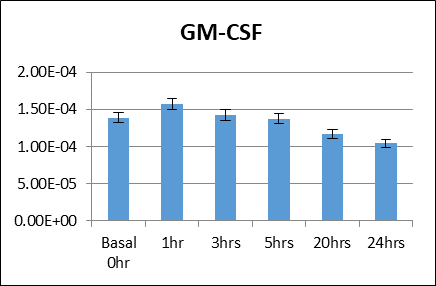

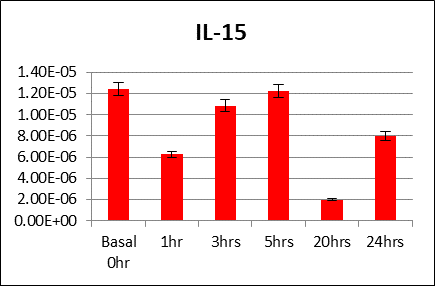

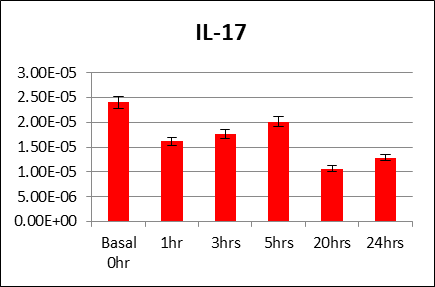

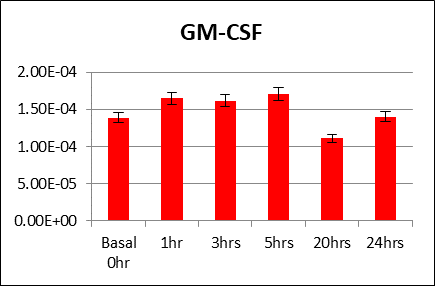


Figure 8. Group 5 Bioplex trend related secreted cytokines IL-15, IL-17 and GM-CSF over a temporal differentiation normalised as pg/cell.

Group 6 (G-CSF, IL-8, VEGF) seen in figure 9 has the most basic trend of all subcategorised cytokines whereby the concentrations is depleted across the board at all time points with minor resurgences detected for VEGF post 5 hrs.


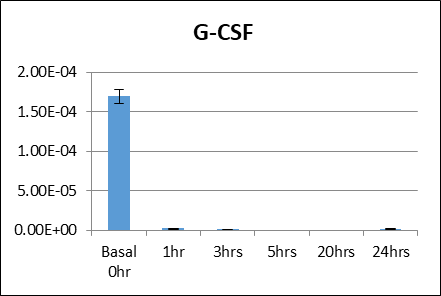

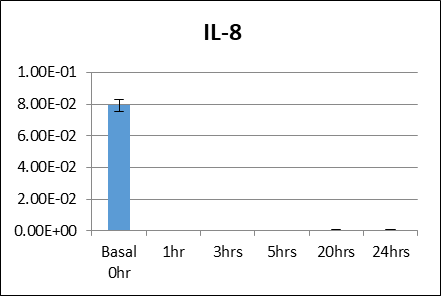

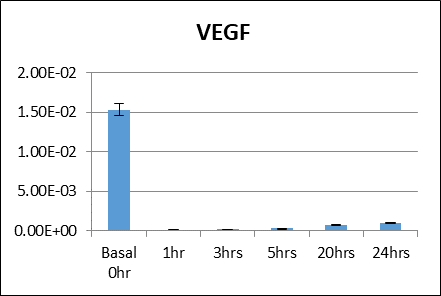

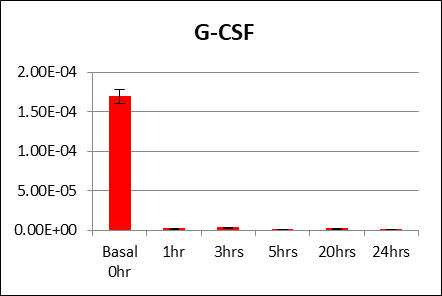

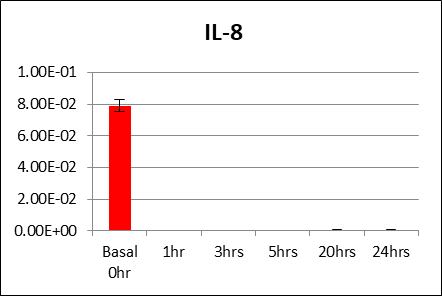

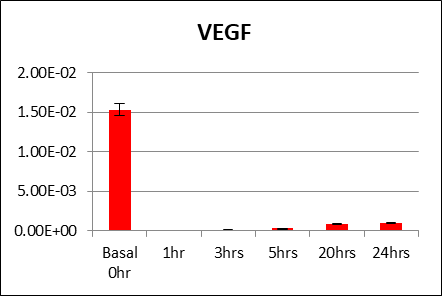


Figure 9. Group 6 Bioplex trend related secreted cytokines G-CSF, IL-8 and VEGF over a over a temporal differentiation normalised as pg/cell.

Group 7 (figure 10) consists of FGF basic and IL-6 which trends are inverse of each other. The FGF basic concentration starts high and subsequently decreases over time whereas the IL-6 starts at undetectable concentrations in the basal cells and increases substantially till the highest concentration at the 24 hour time point.


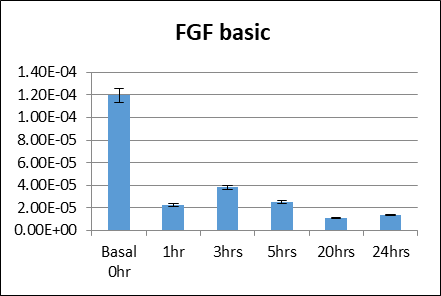

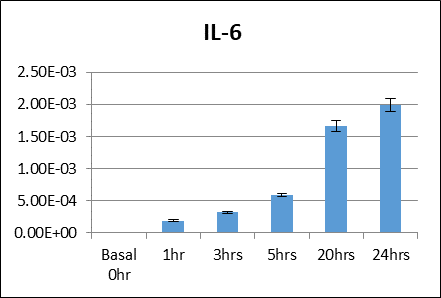

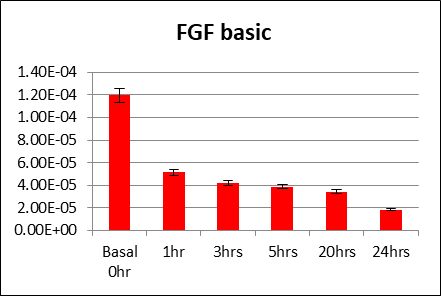

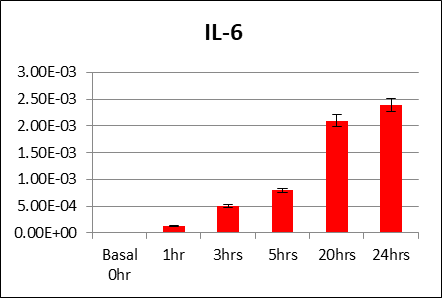


Figure 10. Group 7 Bioplex trend related secreted cytokines FGF-basic and IL-6 over a temporal differentiation normalised as pg/cell (AECK - Blue, LK - Red).

**1D Electrophoresis Experimental Procedure**

Samples were diluted 1:1 with LDS loading buffer (Invitrogen), heated at 95^o^C for 10 minutes then centrifuged. Samples were then loaded into 4-12% Bis-Tris Criterion gel (BioRad) in XT-MES (BioRad) running buffer then electrophoresed according to the standard product protocol of 160V for 50 minutes (BioRad). Upon completion of electrophoresis, unless required for western blot, gels were placed in fix solution (40% methanol and 10% acetic acid) and incubated on a gentle rocker at room temperature for 60 minutes. Gels were then placed in Flamingo fluorescent protein stain (BioRad) and incubated for 60 minutes. Gels were imaged using a PharosFX Plus (Biorad) imager and Quantity One software (BioRad).

**Western blot Experimental Procedure**

The method as per [2], Subsequent to 1D PAGE and transfer, the membrane was then placed in a solution containing the one of the following primary monoclonal antibodies: mouse anti-human NeuN/Fox3 (M377100 Biosensis 1:5000), mouse anti-human NF200 (M988100 Biosensis 1:500), rabbit anti-human βT3 (ab18207 Abcam 1:1000) or rabbit anti-human GFAP (ab7260 Abcam 1:50000) diluted in PBS respectively and incubated overnight at 4oC on a gentle rocker. Subsequently washed 3 times with PBS and probed with a secondary antibody either and anti-mouse

IgG (A4416 Sigma) or anti-rabbit IgG (A4312 Sigma) dependent on the primary probe. Secondary antibodies were peroxidase or alkaline phosphatase conjugated for development with 3, 3-Diaminobenzidine (DAB) (Sigma) or 5-bromo-4-chloro-3-indolyl phosphate/nitro blue tetrazolium chloride (BCIP/NBT) (Sigma) respectively.

**Western Blot Results**

The commonly used neuronal markers βT3, GFAP, NF200 and NeuN were examined within this study as well to compare the expression in the AECK and LK treated ADSCs with GBC whole cell lysates (Figure 11). The βT3 55 kDa protein is strongly detected across all chemically treated ADSCs as well as the positive GBC control. GFAP is highly expressed in the GBCs and in the AECK and LK treated ADSCs have a similar low level of expression detected as the basal ADSCs. The detection of the NF200 protein in the AECK samples presents an equivalent detected level to that seen in the GBCs, while the LK samples show significantly lower intensity and at a comparable level to that seen in the ADSCs. Coincidently, the ADSCs should not be expressing a ‘neurospecific’ marker. This has been is a continual discrepancy in the literature, whereby certain neuronal markers have now been annotated as being expressed in a variety of cells [3-6]. Correspondingly, the discovery of the neuroepithelium specific marker βT3 [7-9] and the minor appearance of NeuN in the ADSCs, AECK and LK samples at a concentration comparatively lower than in the GBCs, contradicts the reported usefulness of these neurospecific markers. This reinforces the value of a proteomic characterisation of cells that have been directed toward a neuronal phenotype with a novel chemical stimulation.


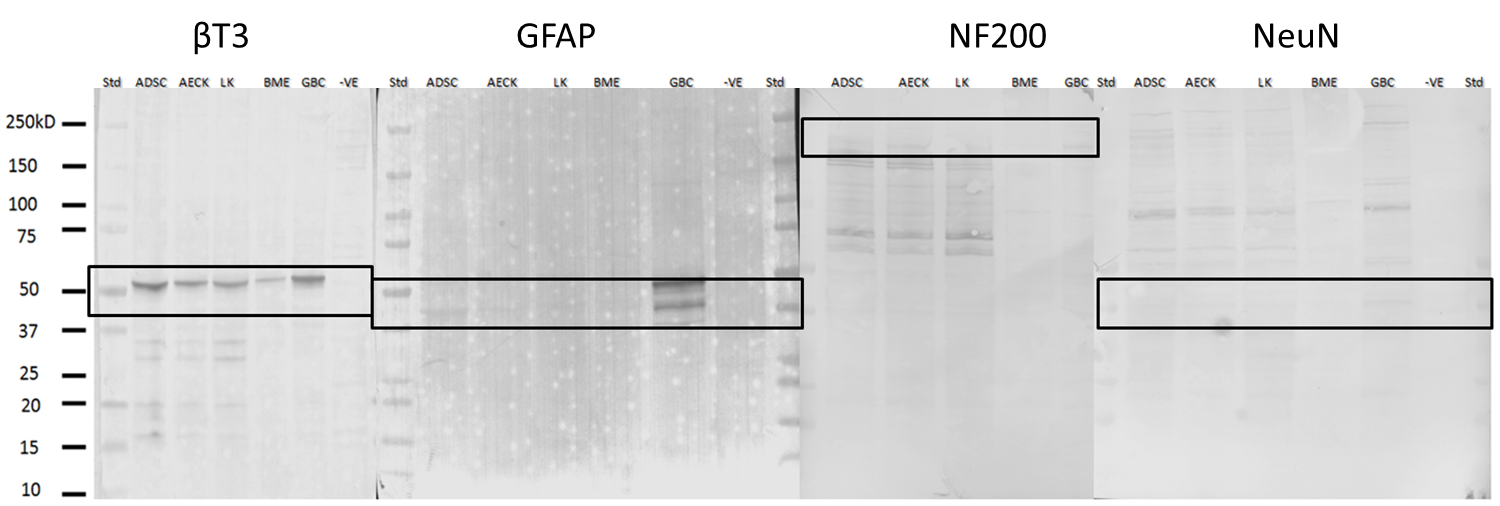


**Figure 11.** Western blot of βT3 positive in ADSCs, AECK, LK, BME differentiated and GBCs seen at 55 kDa. GFAP positively detected in GBCs at 48 kDa and very faintly in chemically treated cells. NF200 positively identified at 200 kDa in GBCs and very weakly in chemically differentiated. NeuN a very low positive in ADSCs, AECK, LK and GBCs only at 46 and 48 kDa. –VE is the negative control of an unrelated cell type.

**Supplementary References**

1. Jobbins, S.E., et al., *Immunoproteomic approach to elucidating the pathogenesis of cryptococcosis caused by Cryptococcus gattii.* J Proteome Res, 2010. **9**(8): p. 3832-41.

2. Santos, J., et al., *Proteomic Analysis of Human Adipose Derived Stem Cells during Small Molecule Chemical Stimulated Pre-neuronal Differentiation.* Int J Stem Cells, 2017.

3. Buniatian, G., et al., *The immunoreactivity of glial fibrillary acidic protein in mesangial cells and podocytes of the glomeruli of rat kidney in vivo and in culture.* Biol Cell, 1998. **90**(1): p. 53-61.

4. Maunoury, R., et al., *Glial fibrillary acidic protein immunoreactivity in adrenocortical and Leydig cells of the Syrian golden hamster (Mesocricetus auratus).* J Neuroimmunol, 1991. **35**(1-3): p. 119-29.

5. Davidoff, M.S., et al., *Leydig cells of the human testis possess astrocyte and oligodendrocyte marker molecules.* Acta Histochem, 2002. **104**(1): p. 39-49.

6. Kasantikul, V. and S. Shuangshoti, *Positivity to glial fibrillary acidic protein in bone, cartilage, and chordoma.* J Surg Oncol, 1989. **41**(1): p. 22-6.

7. Caccamo, D., et al., *Immunohistochemistry of a spontaneous murine ovarian teratoma with neuroepithelial differentiation. Neuron-associated beta-tubulin as a marker for primitive neuroepithelium.* Laboratory investigation; a journal of technical methods and pathology, 1989. **60**(3): p. 390-398.

8. Hoffman, P.N. and D.W. Cleveland, *Neurofilament and tubulin expression recapitulates the developmental program during axonal regeneration: induction of a specific beta-tubulin isotype.* Proceedings of the National Academy of Sciences, 1988. **85**(12): p. 4530-4533.

9. Katsetos, C.D., et al., *Class III beta-tubulin isotype: a key cytoskeletal protein at the crossroads of developmental neurobiology and tumor neuropathology.* J Child Neurol, 2003. **18**(12): p. 851-66; discussion 867.
